# The RNA-binding protein LARP1 is a post-transcriptional regulator of survival and tumorigenesis in ovarian cancer

**DOI:** 10.1093/nar/gkv1515

**Published:** 2015-12-29

**Authors:** Thomas G. Hopkins, Manuela Mura, Hiba A. Al-Ashtal, Roni M. Lahr, Normala Abd-Latip, Katrina Sweeney, Haonan Lu, Justin Weir, Mona El-Bahrawy, Jennifer H. Steel, Sadaf Ghaem-Maghami, Eric O. Aboagye, Andrea J. Berman, Sarah P. Blagden

**Affiliations:** 1 Ovarian Cancer Action Research Centre, Institute of Reproductive and Developmental Biology, Imperial College, London W12 0HS, UK; 2 Department of Biological Sciences, University of Pittsburgh, Pittsburgh, PA 15260, USA; 3 Department of Histopathology, Imperial College Healthcare NHS Trust, London W12 0NN, UK; 4 Imperial College Experimental Cancer Medicine Centre, Division of Cancer, Imperial College Academic Health Science Centre, London W12 0NN, UK; 5 Comprehensive Cancer Imaging Centre, Imperial College, Du Cane Road, London W12 0NN, UK; 6 Department of Oncology, University of Oxford, Old Road, Oxford OX3 7LE, UK

## Abstract

RNA-binding proteins (RBPs) are increasingly identified as post-transcriptional drivers of cancer progression. The RBP LARP1 is an mRNA stability regulator, and elevated expression of the protein in hepatocellular and lung cancers is correlated with adverse prognosis. LARP1 associates with an mRNA interactome that is enriched for oncogenic transcripts. Here we explore the role of LARP1 in epithelial ovarian cancer, a disease characterized by the rapid acquisition of resistance to chemotherapy through the induction of pro-survival signalling. We show, using ovarian cell lines and xenografts, that LARP1 is required for cancer cell survival and chemotherapy resistance. LARP1 promotes tumour formation *in vivo* and maintains cancer stem cell-like populations. Using transcriptomic analysis following LARP1 knockdown, cross-referenced against the LARP1 interactome, we identify *BCL2* and *BIK* as LARP1 mRNA targets. We demonstrate that, through an interaction with the 3′ untranslated regions (3′ UTRs) of *BCL2 and BIK*, LARP1 stabilizes *BCL2* but destabilizes *BIK* with the net effect of resisting apoptosis. Together, our data indicate that by differentially regulating the stability of a selection of mRNAs, LARP1 promotes ovarian cancer progression and chemotherapy resistance.

## INTRODUCTION

Improvements to RNA capture and sequencing methods have highlighted RNA binding proteins (RBPs) as important post-transcriptional contributors to gene expression and cellular behaviour. In normal cells, mRNAs have predetermined half-lives; the most short-lived transcripts being enriched for proto-oncogenic functions such as cell cycle progression and evasion of apoptosis, and those with the longest lifespans encoding housekeeping genes (1–4). There is accumulating evidence that RBPs such as the 5′ cap complex protein eIF4E contribute to human diseases including cancer (1,5) by selectively binding and altering the half-lives of mRNA transcripts involved in pathological processes (6).

La-Related Protein 1 (LARP1) is a highly evolutionarily-conserved RBP and member of the LARP family, each carrying a conserved La domain, an RNA-binding region that was originally identified in La protein (or LARP3/genuine La/SSB (7–11)). LARP1 is unique amongst the LARPs in possessing an additional conserved C-terminal tandem-repeat motif, termed the DM15 region. The crystal structure of this motif has recently been characterized, and identified as a putative mRNA-binding domain (12). LARP1 is a regulator of both mRNA stability and translation (11,13–15), and has recently been shown to bind RAPTOR, act within the mTORC1 signalling cascade and regulate 5′ TOP stability (16,17). LARP1 protein is highly expressed in hepatocellular and lung cancers, where it is an independent predictor of adverse prognosis (18). We have shown previously that expression of LARP1 is elevated in squamous cervical cancer, that LARP1 promotes cell motility and invasion, and is complexed with an mRNA interactome enriched for oncogenic transcripts (15).

Here we explore the role of LARP1 in epithelial ovarian cancer (EOC), a disease responsible for over 140 000 deaths worldwide every year (19). EOC is the most lethal of gynaecological malignancies, with the development of recurrent, increasingly chemotherapy-resistant disease accounting for its high mortality (20,21). Recently, it has been proposed that transformed stem cells may be the origin of some subtypes of EOC and that cancer stem cells are innately chemotherapy resistant (22,23). Here we interrogate the LARP1 interactome in the context of ovarian cancer to characterize the interactions between LARP1 and its target genes and observe the impact of these interactions on stem cell marker expression, chemotherapy resistance and patient survival outcome. Our findings identify LARP1 as a key post-transcriptional regulator of ovarian cancer behaviour.

## MATERIALS AND METHODS

### Cell culture and drug treatment

OVCAR8, HeLa, PEO1, PEO4, IGROV1 and OVCAR4 cells were kindly provided by the Ovarian Cancer Action Biobank at Imperial College, and were genotyped prior to use. SKOV3 and OVCAR3 cells were obtained from ATCC. OVCAR3 cells were cultured in RPMI supplemented with 20% foetal calf serum (FCS) and 0.01 mg/ml bovine insulin (Sigma-Aldritch). All other lines were cultured in RPMI with 10% FCS, with the exception of HeLa cells, which were maintained in Dulbecco's modified Eagle's medium. All media was supplemented with L-glutamine (Gibco) to a final concentration of 2 mM. All lines were cultured at 37°C in 5% CO_2_. For drug treatments, cells were exposed to cisplatin (Accord Healthcare), gemcitabine (Hospira) and paclitaxel (TEVA UK) at the stated concentrations. Salinomycin (Sigma-Aldritch) was resuspended in dimethyl sulfoxide (DMSO) and added to culture medium.

### mRNA-sequencing and data analysis

Total RNA from three biological repeats was extracted from OVCAR8 cells following transient LARP1 knockdown with the miRNeasy kit (Qiagen) following the manufacturer's instructions, with on-column DNAse digestion (QIAGEN). Polyadenylated RNA was enriched using the Dynabead mRNA-purification kit and fragmented using the Ambion fragmentation reagent (both Life Technologies). First-strand cDNA was generated using random hexamer-primed reverse transcription, with First Strand Master Mix and the SuperScript II Reverse Transcriptase kit (Invitrogen), with dUTP used during second-strand synthesis. The resulting cDNA was purified with Agencourt AMPure XP Beads (Beckman Coulter) then end-repaired and 3′ adenylated and adaptors were ligated. Products were separated by agarose gel electrophoresis, and fragments between 300 and 350 bp were excised and eluted. Uracil-N-Glycosylase (UNG, Applied Biosystems) was used to degrade the second-strand cDNA, and products were amplified and re-purified. Library quantification and quality control was performed using the Agilent 2100 Bio-analyzer and the ABI StepOnePlus Real-Time PCR System.

Paired-end 100 bp sequencing was performed using the Illumina HiSeq2000 platform at Beijing Genomics Institute, Shenzhen. Following quality control, clean reads were aligned to Hg19 reference sequences using SOAPaligner/SOAP2 (24), allowing for up to five mismatches. Gene expression was determined using the reads per kilobase per million reads (RPKM) method (25), and the ratio between siCONTROL and siLARP1 samples calculated. Functional enrichment analysis was conducted with Ingenuity Pathway Analysis (QIAGEN), using a change in expression of ±25% and a false discovery rate threshold ≤0.05 to select transcripts.

### RT-qPCR

Total RNA was extracted as before and reverse transcribed using MMLV Reverse Transcriptase (Promega) with random hexamer primers (Promega) according to the manufacturer's instructions. All RT-qPCR assays were performed with exon-spanning TaqMan RNA expression assays (Invitrogen) using Universal Master Mix II (Invitrogen two) on a 7900HT analyser (Applied Biosystems). Treated samples were normalized to controls with the ΔΔC_t_ formula using 18S rRNA as an endogenous control.

### RNA immunoprecipitation (RIP)

Cells were collected by trypsinization and re-suspended in RIP lysis buffer: 20 mM HEPES pH 7.4, 150 mM KCl, 5 mM MgCl2, 0.5% NP40, 400U/ml RNase inhibitor (Promega), 1 mM DTT, 400 μM VRC (NEB), protein (Roche) and phosphatase inhibitor (Calbiochem). Lysates were stored at −80°C overnight. RNA was immunoprecipitated with rabbit anti-LARP1 polyclonal antibody (SDIX-Novus Biologicals) or rabbit IgG isotype control (Cell Signalling Technology) following the method described by Keene *et al*. (26). RNA was extracted with Trizol (Life Technologies) and purified with the RNA clean-up and concentration micro kit (Norgen Biotek). To generate cDNA, immunoprecipitated RNA was reverse transcribed using the SensiScript^®^ RT Kit (Qiagen) following the manufacturer's instructions. RT-qPCR was performed as described earlier. The fold enrichment for each target was measured by comparing the Ct values of LARP1-immunoprecipitated fraction to the IgG isotype fraction and normalized using the ΔC_t_ formula.

### Protein extraction and western blotting

Cells were washed and incubated with protein lysis buffer (1% NP-40, 10 mM Tris–HCl pH 7.5, 150 mM NaCl, with protease and phosphatase inhibitors as before) for 10 min on ice. Lysates were cleared by centrifugation and protein was quantified using the microBCA protein assay kit (Thermo Scientific). Protein samples were boiled with Laemmli buffer and separated by sodium dodecyl sulphate-polyacrylamide gel electrophoresis (SDS-PAGE). Proteins were transferred to nitrocellulose membranes. Blocking and antibody incubation was performed according to antibody manufacturers’ instructions with anti-LARP1 (rabbit, SDIX-Novus Biologicals), anti-BCL2 (mouse, Santa Cruz), anti-BIK (goat, Santa Cruz) and anti-HSP60 (rabbit, Abcam) antibodies. Appropriate HRP-conjugated secondary antibodies were obtained from Dako.

### Transfection and transduction

Plasmids containing shGFP (TR30016) and shLARP1 (TF303581D) sequences, the Flag-BCL2 overexpression plasmid and matched, empty vector control were all purchased from Origene. Cells were transfected using Effectene (Qiagen), as per manufacturer's instructions, and selected with 2 μg/ml puromycin after 48 h. (Sigma-Aldrich). To create lentiviral-transduced lines, cells were infected with virus and selected with 2 μg/ml puromycin after 24 h. Lentiviruses and Mission shRNA constructs were obtained from Sigma-Aldrich (Control [SHC0016] and shLARP1 [TRCN0000150984, TRCN0000152624, TRCN0000152891]).

For transient knockdown, sub-confluent cells were transfected using Dharmafect 1 (GE Dharmacon) as previously described (27), with control non-targeting siRNA (GGUCCGGCUCCCCCAAAUG) or LARP1-targeting siRNA (GAAUGGAGAUGAGGAUUGC, AGACUCAAGCCAGACAUCA) synthesized by Eurofins, or BCL2-targeting siRNA (D-003307–02) obtained from GE Dharmacon.

### Luciferase UTR reporter assays

The 3′ UTR sequence for BCL2 and the 3′ and 5′ UTR sequences for BIK were obtained from the UCSC Genome Browser (28). The sequence of the 203 bp BCL2-ARE region was as described by Ishimaru *et al*. (29). Sequences were cloned into 3′ and 5′ UTR *renilla* luciferase reporter constructs (SwitchGear Genomics). β-actin 5′ UTR cloned into a renilla luciferase reporter construct was used as a negative control. Cells were co-transfected with a *firefly* luciferase control plasmid using Effectene as before. *Renilla* and *firefly* luciferase activity was analysed using the Dual-luciferase reporter assay system (Promega) in triplicate in 96-well plates with luminescence recorded using the LUMOstar Optima plate reader (BMG Labtech). *Renilla* luminescence was normalized to *firefly* activity and control, and LARP1 knockdown samples compared. For luciferase mRNA analysis, total RNA extraction, with on-column DNAase-digestion and cDNA production was performed as described above. Custom *renilla* and *firefly* luciferase mRNA TaqMan assays were obtained from Invitrogen Life Technologies.

### LARP1 purification

Full length LARP1 (amino acids 1–1019) was cloned into a modified pET28 vector that expressed an N-terminal 6×HIS-MBP LARP1 fusion protein. Protein was expressed using autoinduction of BL21(DE3) cells for 3 h at 37°C and then 18°C overnight. A total of 15 g of cells were lysed the same day as harvesting using homogenization in lysis buffer (50 mM Tris pH 7.5, 500 mM NaCl, 30 mM imidazole, 10% glycerol). Protein was purified using batch binding of 5 ml of HisPur Ni-NTA Resin (ThermoScientific) and eluted in lysis buffer with the addition of 250 mM imidazole. The N-terminal 6×HIS-MBP tag was cleaved overnight using TEV cleavage and the protein was further purified from contaminants and nucleic acid using tandem anion and cation exchange and size exclusion chromatography.

### Electrophoretic mobility shift assays (EMSAs)

RNA was transcribed *in vitro* at 37°C for 14 h using T7 RNA polymerase and gel purified. RNAs were 5′ end labelled with [γ-^32^P]ATP (Perkin Elmer) using T4 polynucleotide kinase (NEB) and gel purified again. Each binding reaction contained the indicated final concentration of recombinant LARP1, five hundred counts of radiolabelled RNA (<2 nM final concentration), binding buffer (20 mM Tris–HCl, pH 8, 150 mM NaCl, 10% glycerol, 1 mM DTT), 1 μg bovine serum albumin (BSA) and 0.5 μg tRNA. Reactions were incubated on ice for 30 min and analysed on an 8% polyacrylamide (29:1) native 0.5× tris-glycine gel with a 5% stacking gel at 4°C. Gels were dried, exposed overnight to phosphor screens and scanned with a Fuji plate reader.

**RNA sequences:**

BIK construct 1:

UGACCACUGCCCUGGAGGUGGCGGCCUGCUGCUGUUAUCUUUUUAACUGUUUUCUCAUGA

UGCCUUUUUAUAUUUAAACCCCGAGAUAGUGCUGGAACACUGCUGAGGU;

BIK construct 2: GUGCUGGAACACUGCUGAGG

UUUUAUACUCAGGUUUUUUGUUUUUUUUUUAUUCCAGUUUUCGUUUUUUCUAAAAGAUGA

AUUCCUAUGGCUCUGCAAUUGUCACCGGUU

AACUGUGGCCUGUGCCCAGGAAGAGCCAUUCACUCC;

BIK construct 3: GGAAGAGCCAUUCACUCCUG

CCCCUGCCCACACGGCAGGUAGCAGGGGGAGUGCUGGUCACACCCCUGUGUGAUAUGUGAUG

CCCUCGGCAAAGAAUCUACUGGAAUAGAUUCC

GAGGAGCAGGAGUGCUCAAUAAAAUGUUGGUUUCCAGC.

BCL2 construct 1: GGCUCCACCUGGAUGUUCUGUGCCUGUAAACAUAGAUUCGCUUUCCAUGU

UGUUGGCCGGAUCACCAUCUGAAGAGCAGACGGAUGGAAAAAGGACCUGAUCAUUGGGGAAG

CUGGCUUUCUGGCUGCUGGAGGCUGGGGAGAAGGUGUUCAUUCACUUGCAUUUCUUUGCCCUGGG;

BCL2 construct 2: GCCCUCCUGCCCUCCUUCCGCGGGGGCUUUCUCAUGGCUGUCCUUCAGGG

UCUUCCUGAAAUGCAGUGGUGCUUACGCUCCACCAAGAAAGCAGGAAACCUGUGGUAUGAAGCCAGACCUCCCCGG;

BCL2 construct 3: GCCUGUUUUGUCUUUUGUUGUUGUUCAAACGGGAUUCACAGAGUAUUU

GAAAAAUGUAUAUAUAUUAAGAGGUCACGGGGGCUAAUUGCUGGCUGGCUGCCUUUUGCUGUGGGGUUUUGUU;

BCL2 construct 4: GCCCUCCAGAUAGCUCAUUUCAUUAAGUUUUUCCCUCCAAGGUAGAAUUU

GCAAGAGUGACAGUGGAUUGCAUUUCUUUUGGGGAAGCUUUCUUUUGGUGGUUUUGUUUA

UUAUACCUUCUUAAGUUUUCAACCAAGGUUUGCUUUUGUU.

OAZ1:

GGCCGCCUCGGGGCUGGGCAUCCGGCCCCUGGGGCCACCCCUUGUCAGCCGGGUGGGUAG

GAACCGUAGACUCGCUCAUCUCGCCUGGGUUUGUCCGCAUGUUG.

RPS6:

CCUCUUUUCCGUGGCGCCUCGGAGGCGUUCAGCUGCUUCAAG.

BIK 5′ UTR:

GCAGACACGAAGCCUCCCGGGUGGCUUACAGACGCUGCCAGCAUCGCCGCCGCCAGAGGAGA;

BIK CDS1:

GUCUGAAGUAAGACCCCUCUCCAGAGACAUCUUGAUGGAGACCCUCCUGUAUGAGCAGCUCC

UGGAACCCCCGACCAUGGAGGUUCUUGGCAUGACUGACUCUGAAGAGGACCUGGACCCUA

UGGAGGACUUCGAUUCUUUGGAAUGCAUGGAGGGCAGUGACGCAUUGGCCCUGCGGCUGGC;

BIK CDS2:

GAGGACAUCAGGGAUGUUCUUAGAAGUUUCAUGGACGGUUUCACCACACUUAAGGAGAACAU

AAUGAGGUUCUGGAGAUCCCCGAACCCCGGGUCCUGGGUGUCCUGCGAACAGGUGCUGCU

GGCGCUGCUGCUGCUGCUGGCGCUGCUGCUGCCGCUGCUCAGCGGGGGCCUGCACCUGCUGCUC.

### Competition assays

RPS6 (42mer) was annealed in 1× binding buffer at 95°C for 1 min and snap cooled. RPS6 was prebound to 250 nm LARP1 for 30 min at 4°C in binding buffer, 0.5 μg TRNA and 1 μg BSA. Prebound LARP1 was competed off with 5× cold RNA stocks yielding a final concentration of 1, 10, 30 μM of RPS6, BCL2 or OAZ1. Reactions were incubated on ice for 30 additional minutes and 8 μl was then loaded on a 0.5× TBE gel, run for an hour at 120V, dried and exposed overnight.

### Immunofluorescence (IF) staining and confocal imaging

Cells (SKOV3) were cultured on glass coverslips for 24 h. Cytoplasmic mRNP granule formation was triggered by treatment with 5 μg/ml sodium arsenite (Sigma-Aldrich) for one hour. Cells were washed before being incubated with PHEM fixative (4% PFA, 60 mM PIPEs, 25 mM HEPES, 10 mM EGTA, 4 mM MgCl_2_) for 10 min. Fixative was removed and cells were washed and blocked in PBSTB buffer (1% BSA, 0.1% TritonX-100) for 1 h. Cells were stained with antibodies to DCP1A (Abnova), PABP (Abcam) and LARP1 (SDIX- Novus Biologicals). Primary antibody solution was applied and incubated overnight at 4°C. After washing, Alexa Fluor-conjugated secondary antibodies (Life Technologies) were applied and incubated at room temperature for 1 h. Cells were washed and mounted with ProLong Gold mounting medium with DAPI (Life Technologies). Immunofluorescence staining was analysed using Leica 500 confocal microscope and images processed with Leica LAS AF lite software.

### 3-(4,5-Dimethlythiazol-2-YI)-2,5-Diphenyltetrazolium Bromide (MTT) viability and caspase activity assays

For MTT labelling, cells were cultured at 37°C in 96-well plates with 100 μl media and labelled with 20 μl of MTT (3 mg/ml, Sigma-Aldrich) for 1 h. Precipitate was solubilized overnight with 10% SDS in 0.01M HCl. Absorbance at 570 nm was recorded on an OPTImax microplate reader (Molecular Devices). MTT assays were performed at the timepoints specified in figure legends. Caspase 3/7 activity was assessed using the CaspaseGlo-3/7 Assay (Promega). Cells were cultured at 37°C in white 96-well plates (Corning). CaspaseGlo reagent was added to each well and plates left at room temperature for 1 h before reading on a LUMOstar Optima plate reader (BMG Labtech). Assays were performed at 24 h, unless otherwise stated.

### Xenograft experiments

All animal experiments were performed by licensed investigators in accordance with the United Kingdom Home Office Guidance on the Operation of the Animal (Scientific Procedures) Act 1986 and within the published guidelines for the welfare and use of animals in cancer research (30). Female SCID-Beige or NOD-SCID IL2R-gamma^null^ (NSG) mice (aged 6–8 weeks; Charles River) were used. SKOV3 cells (2 × 10^6^ unless otherwise specified) were injected subcutaneously into the flanks of mice (at least five per cohort). For limiting dilution experiments, cells were diluted 1:1 in phenol-free growth factor-reduced Matrigel (BD Biosciences) prior to implantation. Tumour dimensions were measured using electronic callipers and tumour volumes calculated by the equation: volume = (π/6) × a × b × c, where a, b and c represent three orthogonal axes of the tumour. Tumours were classed as measurable when they reached ≥5 mm in any axis. Experiments were terminated at 2 months, or before any mouse reached pre-set welfare limits. Tumours were collected and immediately fixed in 10% formalin for 48 h before paraffin embedding and sectioning.

### Clonogenic assays

Single-cell suspensions of cells were seeded in 10 cm plates, with 1–5 × 10^3^ cells per plate and incubated for 2 weeks. Colonies formed were fixed in ice-cold methanol and stained with 0.5% crystal violet. Plates were photographed using a GE ImageQuant LAS 4000 and colonies were counted with ImageJ.

### Flow cytometry

For cell cycle analysis, cells were trypsinized and fixed in ice-cold 75% ethanol overnight, before RNA digestion (RNAse A, 100 μg/ml) and propidium iodide staining (25 μg/ml, both Sigma-Aldritch). Samples were analysed on a FACSCalibur (BD Biosciences). Cell cycle distribution was determined using FlowJo software (FlowJo LLC). For assessment of apoptosis, cells were resuspended in Annexin V-binding buffer (BioLegend) and incubated for 10 min with Annexin V-FITC antibody (IQ Products). Cells were then washed twice and, following addition of propidium iodide, analysed as before. Assessment of CD133 membrane positivity was performed using CD133/1-APC and IgG isotype-APC (both MACS) and the Aldefluor assay was performed as per manufacturer's instructions (Stemcell Technologies).

### BCL2 promoter activity assay

A BCL2 promoter construct containing P1 and P2 elements (ATG to −3934) upstream of *firefly* luciferase (31) was obtained from Addgene (plasmid LB332). Transient LARP1 knockdown was performed as before. Effectene was used to introduce the BCL2 promoter construct together with a *renilla* luciferase control for data normalization. Twenty-four hours after transfection, total RNA was collected and relative levels of *firefly* and *renilla* luciferase mRNA were determined by RT-qPCR as described above.

### Gene expression array data

Expression data for LARP1 was obtained from Oncomine (www.oncomine.org) for three independent datasets (TCGA (32), Hendrix *et* *al*. (33), Bonome *et* *al*. (34)). Fold change was calculated as median-centered intensity of each cancer sample divided by the mean of non-cancer samples. Significance was calculated using the Student *t*-test. Survival association was determined by Cox regression analysis using the survival package of R. Progression-free survival (PFS) data in ovarian cancer and overall survival data in breast cancer were obtained from *kmplot.com* (35,36). Array gene expression data for PROM1 in the NCI60 panel was obtained from the CellMiner online tool (37).

### Immunohistochemistry

A tissue microarray (TMA) containing normal ovarian tissue (*n* = 40) and ovarian cancer cores (*n* = 40) was obtained from US Biomax (OV801). Other TMAs were developed in Imperial College as detailed in Results. Paraffin was removed with Histoclear and sections were rehydrated in graded alcohols and heated in a microwave oven at 900W for 15 min in citrate buffer pH 6. Tissue sections were pretreated using 0.3% H_2_O_2_ in phosphate bufferedsaline (PBS), rinsed in PBS and then incubated with 20 μl/ml normal goat serum. Primary antibodies, anti-LARP1 (SDIX-Novus Biologicals) or anti-Ki67 antibody (Leica) were diluted in PBS and incubated overnight at 4°C. Secondary or biotinylated-secondary antibodies were incubated for 30 min at room temperature and then processed with the Polymer-HRP Kit (BioGenex), or stained using the Vectastain Elite ABC Kit (Vector labs) and ImmPACT DAB (Vector labs). Tissues were then counterstained with haematoxylin. Intensity of staining was defined for each specimen (0–3, with 3 being most intense) multiplied by the percentage of cells stain-positive (to give a total score out of 300). All images were captured using a Nikon Eclipse ME600. Xenograft samples were processed in the same manner as clinical TMAs.

### LARP1 ELISA

Nunc MaxiSorp plates (Thermo Scientific) were coated overnight at 4°C with anti-LARP1 antibody (mouse, Abnova). Plates were blocked with casein solution (Pierce) then incubated with patient samples diluted 1:20 in AD3 assay diluent (Neuromics). Serial dilutions of recombinant LARP1 protein (Abnova) formed the standard curve. Plates were washed with PBST then incubated with anti-LARP1 antibody (rabbit, SDIX – Novus Biologicals) diluted in PBST. Following a further wash in PBST, plates were incubated with peroxidase-conjugated goat anti-rabbit secondary antibodies (Dako) diluted in PBST. Plates were developed using Luminata Forte ELISA HRP substrate (EMD Millipore) and read using a LUMOstar Optima plate reader (BMG Labtech).

### Statistical analysis

Statistics analyses were performed using GraphPad Prism software (GraphPad Software Inc.), unless otherwise stated. Statistical tests appropriate to the experiment were chosen as indicated in figure legends (Student *t*-test, Chi-Squared, Log-Rank). *P* ≤ 0.05 was taken to be statistically significant.

### Study approval

Tissue and plasma samples were provided by the Ovarian Cancer Action Biobank, supported by Ovarian Cancer Action and Imperial College Healthcare NHS Trust Tissue Bank, supported by the National Institute for Health Research (NIHR) Biomedical Research Centre and based at Imperial College Healthcare NHS Trust and Imperial College London. Informed consent was taken from all patients prior to sampling. Study approval was obtained from the local Research Ethics Committee.

## RESULTS

### Transcriptomic analysis on LARP1 knockdown identifies alterations in the expression of survival-related genes

**Figure 1.**
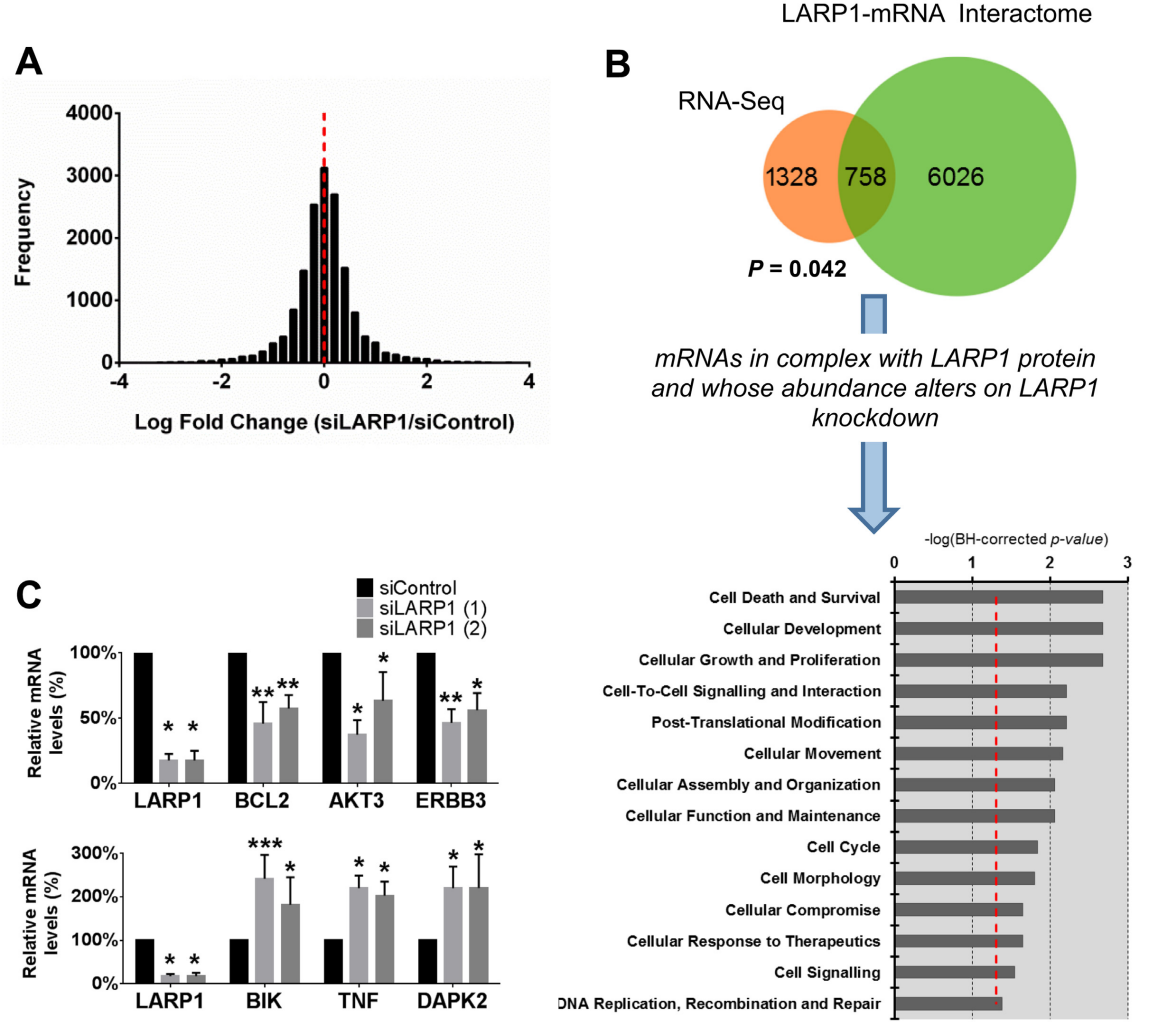
LARP1 knockdown causes alteration in the cancer cell transcriptome. (**A**) Frequency distribution of Log_2_-fold change in transcript abundance in OVCAR8 cells following transient LARP1 knockdown (LARP1 knockdown relative to control). (**B**) Venn diagram showing the overlap between genes with altered mRNA abundance on LARP1 knockdown, with the published LARP1–mRNA interactome, together with the *P*-value for the hypergeometric probability of this overlap, calculated using the R dhyper function (based on 2086 differentially expressed mRNAs and an mRNA interactome with 6784 members) (15). Of the 758 genes present in both datasets molecular ontology enrichment with Ingenuity Pathway Analysis (IPA) was performed (-log[BH-corrected *P*-value] shown, bold dashed line indicates *P* = 0.05). (**C**) RT-qPCR validation of fold changes in mRNA abundance of key survival-associated genes identified from the IPA enrichment analysis following LARP1 knockdown. ***P < 0.001, **P < 0.01, *P < 0.05. Student *t*-test. Minimum of three experimental repeats. Error bars indicate SEM.

In a previous systematic screen of LARP1 expression in diverse malignancies, we found that the protein was highly expressed in several different cancer types, including EOC (15). To explore the role of LARP1 in the ovarian cancer cell, we performed mRNA-sequencing following LARP1 knockdown in the malignant ovarian OVCAR8 cell line. Data from three biological replicates were combined. In control cells, *LARP1* was in the top 7% of the most abundant mRNAs, with transcript abundance comparable to translational components such as *EIF4A3* and *RPL36A*, and together with SSB, was the most highly expressed LARP family member (Supplementary Figure S1A). Following LARP1 knockdown, an equal number of mRNAs displayed increased and decreased abundance (Figure 1A). Ingenuity gene ontology analysis revealed transcripts with altered levels on LARP1 knockdown were significantly enriched for functions linked to cancer (Supplementary Figure S1B). Molecular and cellular function analysis showed enrichment for genes associated with cell growth and proliferation, and cell death and survival (Supplementary Figure S1C).

In order to identify transcripts whose stability is potentially regulated by LARP1, we cross-referenced our RNA-seq data with the previously published LARP1–mRNA interactome, derived from a LARP1 RNA-immunoprecipitation and expression array analysis (RIP-Chip) experiment performed in HeLa cells (15). Although these data were obtained from a cell line derived from a different gynecological malignancy, we hypothesized that protein–mRNA interactions would be sufficiently conserved to yield useful trends. Indeed, we found genes with altered expression after LARP1 knockdown in OVCAR8 cells were more likely to be represented in the LARP1–mRNA interactome (hypergeometric probability distribution, *P* = 0.042), suggesting that interactions between LARP1 protein and target mRNAs could control their abundance (Figure 1B). Of the 758 genes both differentially expressed on LARP1 knockdown and in complex with LARP1 protein, 49% showed decreased transcript abundance on LARP1 knockdown, whilst 51% showed increased abundance, suggesting LARP1 may be capable of both stabilising and destabilising transcripts. Functional enrichment analysis of these 758 genes revealed that the regulation of cell survival was the most significant biological trait (Figure 1B). Following LARP1 knockdown, mRNA levels of pro-survival genes such as *BCL2, ERBB3* and *AKT3* were significantly reduced while expression of pro-apoptotic genes, including *BIK, TNF* and *DAPK2* were increased. To validate the RNA-seq findings, we repeated LARP1 knockdown with two independent siRNAs and confirmed changes in expression of these genes with RT-qPCR (Figure 1C).

These data demonstrate LARP1 knockdown has a marked effect on the cancer cell transcriptome and alters the expression of key apoptosis-related genes.

### LARP1 is a component of BCL2 messenger ribonucleoprotein (mRNP) complexes and promotes BCL2 transcript stability

**Figure 2.**
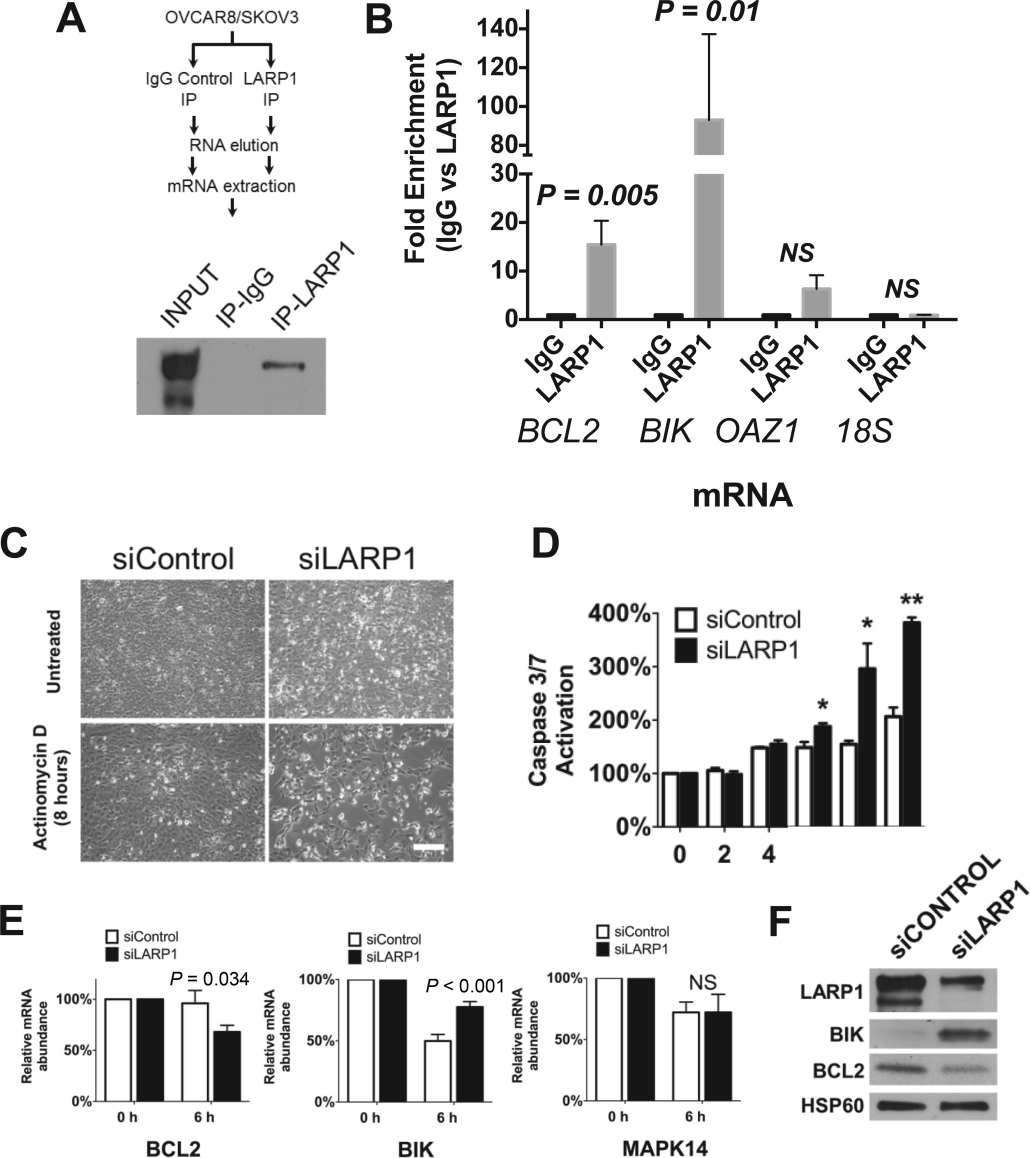
LARP1 is a component of BCL2-containing mRNP complexes and promotes transcript stability. (**A**) Schematic of LARP1 RNA-immunoprecipitation (RIP) with representative western blot of LARP1 protein following LARP1-immunoprecipiation in OVCAR8 and SKOV3 cells. (**B**) RT-qPCR analysis of BCL2 and BIK mRNA obtained from RIP with anti-LARP1 and IgG control antibodies in OVCAR8 cells. OZA1 and 28S were included as negative controls. (**C**) Representative cell images following LARP1 knockdown and 8 h exposure to actinomycin D (Scale bar 200 μm). (**D**) Following transient knockdown of LARP1, OVCAR8 cells were treated with actinomycin D to halt transcription and apoptosis (as determined by cleaved Caspase 3/7) was monitored using the CaspaseGlo assay (data normalized to *T* = 0, at the time of actinomycin-D administration). **P < 0.01, *P < 0.05. (**E**) Stability of BIK and BCL2 mRNA following treatment with actinomycin D for 6 h. Relative abundance was determined by RT-qPCR (ΔΔCt). MAPK14 was chosen as a negative control as its mRNA abundance did not alter on LARP1 knockdown in the RNA-seq dataset. Student *t*-test. Minimum of three experimental repeats. Error bars indicate SEM. (**F**) Western blotting of BIK and BCL2 protein levels following LARP1 knockdown.

The positive and highly significant association between LARP1 expression and *B-cell lymphoma 2* (*BCL2*) mRNA transcript abundance was of particular interest. BCL2 is an important anti-apoptotic oncoprotein, known to promote chemotherapy resistance in ovarian cancer cells and to be associated with patient outcomes (38–41). By contrast, *BIK* encodes a pro-apoptotic protein. To confirm LARP1 protein interacts with *BCL2* and *BIK* mRNAs, as suggested by the published LARP1 interactome (15), we performed RNA-immunoprecipitation in OVCAR8 and SKOV3 ovarian cancer cell lines (Figure 2A). OAZ1 and 18S were used as controls for this and subsequent experiments as they are absent from the LARP1-interactome (15). Both *BCL2* and *BIK* transcripts were highly enriched in anti-LARP1 immunoprecipitates when compared to the *OAZ1* and *18S* in which no significant fold-enrichment was observed (Figure 2B). These data confirmed that *BCL2* and *BIK* co-associate with LARP1 in mRNP complexes.

As LARP1 has been identified as a regulator of mRNA-stability (13–15), and BCL2 expression is known to be post-transcriptionally regulated (29), we investigated whether the changes in transcript abundance of *BCL2* and *BIK* following LARP1 knockdown were due to an effect on their stability. To assess this, we knocked-down LARP1 and treated cells with actinomycin D to halt transcription. LARP1 knockdown resulted in significantly increased apoptosis, when compared to control cells, (Figure 2C and D), supporting the importance of LARP1 in the post-transcriptional regulation of cell survival. We assessed mRNA abundance at 6 h and observed a significant decrease in *BCL2* transcript levels in LARP1 knockdown cells when compared to controls, demonstrating that LARP1 is required to maintain the stability of *BCL2* transcripts. The opposite trend was observed for *BIK*, where LARP1 knockdown was associated with an increase in *BIK* mRNA stability (Figure 2E). The same trend was observed on western blotting (Figure 2F). In contrast, there was no significant change in the transcript stability of *MAPK14*, a negative control. From this we concluded that LARP1 exerts a binary effect on BCL2 and BIK, stabilising BCL2 whilst destabilising BIK mRNAs, with the net effect of resisting apoptosis.

### LARP1 regulates target stability at the level of the 3′ UTR

**Figure 3.**
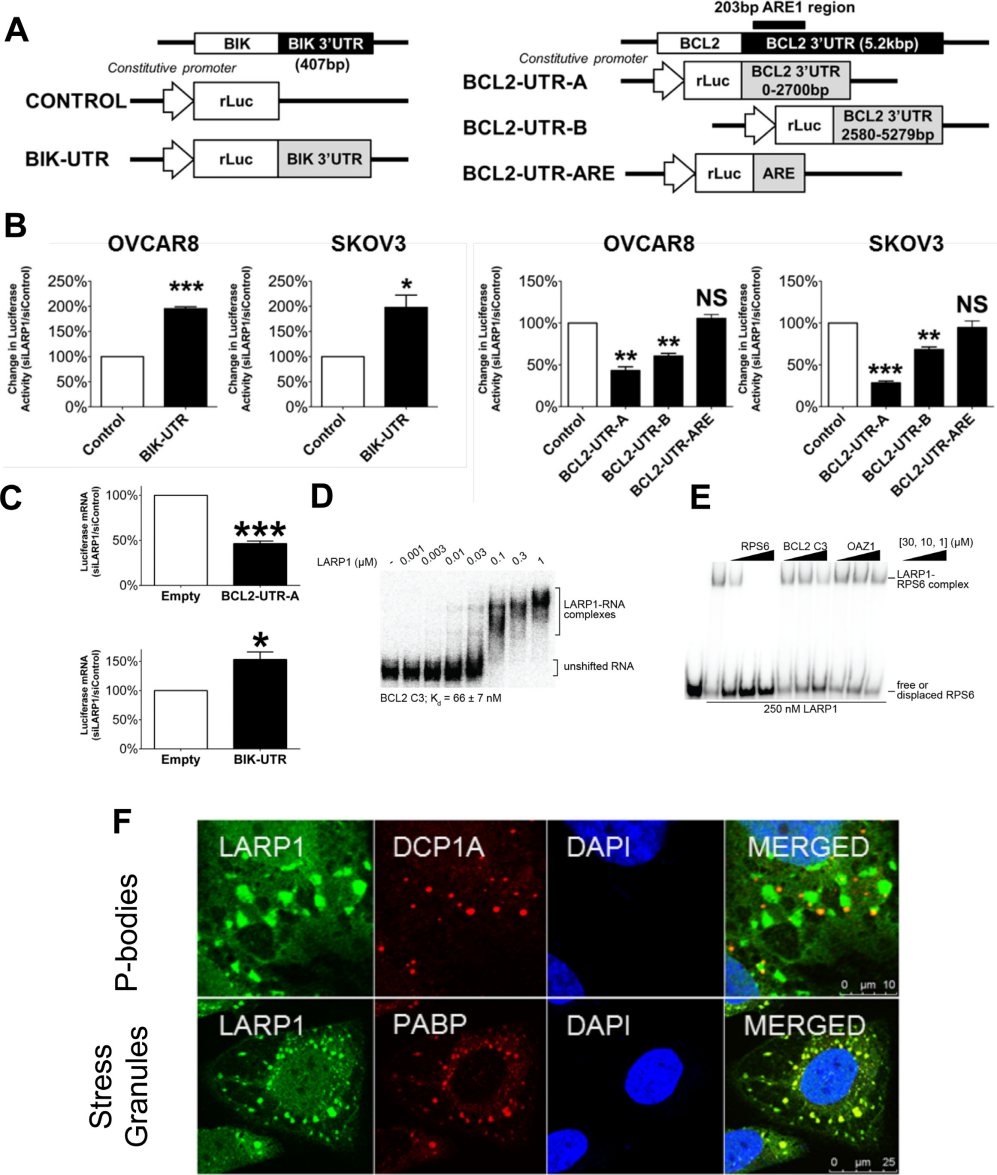
LARP1 regulates stability at the level of the 3′ UTR and binds the BCL2 3′ UTR via its DM15 domain. (**A**) Schematics outlining construction of 3′-untranslated region (3′ UTR) reporter constructs for BIK and BCL2. Both were compared relative to a control vector with no additional 3′ UTR sequence. (**B**) OVCAR8 and SKOV3 cells were co-transfected with *Renilla* luciferase 3′ UTR constructs and a control *Firefly* luciferase control vector. *Renilla* luciferase activity following LARP1 knockdown was determined for each 3′ UTR construct cells. Data was normalized to *Firefly* luciferase activity. (**C**) OVCAR8 cells were co-transfected with *Renilla* luciferase 3′ UTR constructs and a control *Firefly* luciferase control vector and *Renilla* luciferase mRNA abundance following LARP1 knockdown was determined using RT-qPCR. (**D**) Electrophoretic mobility shift assay (EMSA) of LARP1 with a fragment of the BCL2 3′ UTR (construct 3). The affinity of this interaction is indicated. (**E**) Competition experiment analysed by EMSA of LARP1 pre-bound to radiolabelled RNA representing the 5′ TOP of RPS6 and competed with cold competitors, as indicated. (**F**) Confocal immunofluorescence microscopy of SKOV3 cells treated with sodium arsenite to trigger aggregation of mRNP bodies. Cells were stained for LARP1 protein (green) and either the P-body marker DCP1a or stress granule marker PABP (both red). Scale bar 10 μm (top) and 25 μm (bottom).

The 3′-and 5′ untranslated regions (3′ UTRs) of mRNAs contain regulatory elements that determine their stability (42). We hypothesized that the effect of LARP1 on *BCL2* and *BIK* stability may be dependent on sequences present in their UTRs. *BIK* transcripts have a 407 nt 3′ UTR, whilst the *BCL2* 3′ UTR is 5.2 knt long. We designed reporter constructs with 3′ UTR sequences of both genes downstream of the *renilla* luciferase gene (Figure 3A). OVCAR8 and SKOV3 cells were co-transfected with a *firefly* luciferase plasmid to allow data normalization. In both cell lines LARP1 knockdown resulted in a significant increase in luciferase activity in the *BIK 3*′ *UTR* construct when compared to the empty vector control, suggesting LARP1 destabilizes *BIK* mRNA in 3′ UTR-dependent manner (Figure 3B). We then created two equal-sized, overlapping constructs, spanning the length of the *BCL2* 3′ UTR (*BCL2-UTR-A* and *BCL2-UTR-B*). Addition of either *BCL2* 3′ UTR sequence (*A* or *B*) resulted in a significant decrease in luciferase signal on LARP1 knockdown, with a greater effect on stability seen for the proximal 3′ UTR construct (*BCL2-UTR-A*). This stop codon-proximal region contains a 203 nt sequence, termed the *BCL2-*ARE, with multiple AU-rich elements (AREs), pentamer motifs that are important in determining transcript stability (42). This region has previously been implicated in the regulation of *BCL2* mRNA stability (29). We created a third construct containing only this 203 nt sequence (*BCL2-ARE*). Following LARP1 knockdown, there was no significant change in luciferase activity in this construct compared to the control plasmid (Figure 3B), suggesting that the LARP1-mediated stability effect is dependent on additional sequences outside this well-characterized region. Similar trends were observed when analysing luciferase transcript abundance with RT-qPCR (Figure 3C), confirming changes were due, at least in part, to effects on mRNA stability.

As recent work has suggested a role for LARP1 in regulating the translation and stability of TOP-bearing mRNAs via their 5′ UTRs (12,16,17), we investigated whether LARP1 could mediate transcript stability via the 5′ UTR, as well as the 3′ UTR of its target genes. We performed the luciferase assay, as described above but using a 5′ luciferase reporter system containing BIK 5′ UTR or the 5′ UTR of a control (β-actin) in OVCAR8 and SKOV3 cells. Following LARP1 knockdown, there was no significant change in luciferase activity in the two constructs (Supplementary Figure S1D). In line with our previous findings (15), these data suggest that LARP1 differentially regulates mRNA stability through associations with the 3′ UTR of target mRNAs.

To identify potential sites of interaction between LARP1 and the 3′ UTR of target transcripts, we created approximately equal length fragments of the entire BIK 3′ UTR and four similar sized RNA constructs of the pyrimidine-rich regions of the BCL2 3′ UTR, sequences with which the DM15 has been shown to interact (12). We then analysed binding by whole length LARP1 protein (Supplementary Figure S2A and S2B). We observed an interaction between LARP1 and all four pyrimidine-rich fragments of the BCL2 3′ UTR tested. LARP1 interacted with construct 3 with ∼4× higher affinity (Supplementary Figure S2B and Figure 3D). The interactions between LARP1 and these constructs was maintained by the DM15 region of LARP1 (data not shown). We also tested the 3′ UTR of OAZ1, which is a sequence that was not identified in the LARP1 interactome (15), two regions in the coding sequence of BIK and the 5′ UTR of BIK for binding to LARP1; none of these bound with affinity tighter than 250 nM (Supplementary Figure S2C).

To examine the specificity of these interactions, we conducted competition assays using the 5′ UTR from RPS6, which we have shown to bind the DM15 motif of LARP1 specifically (12). As judged by radiolabelled RPS6 that was displaced by cold competitor RNA, these experiments demonstrated that LARP1 specifically binds the BCL2 construct 3, but not the negative control, the OAZ1 3′ UTR (Figure 3E). Thus, the binding of LARP1 to BCL2 constructs 1, 2 and 4 and to the BIK constructs seems to be non-specific. Since the LARP1 protein used in these experiments was expressed and purified from *Escherichia coli*, it is possible that post-translational modifications are required to tune the binding of LARP1 to the BIK 3′ UTR.

LARP1 has previously been found in stress granules, sites of mRNA storage and P-bodies, sites of RNA degradation (14,43). As LARP1 appeared to be capable of both positively and negatively regulating transcript stability, we were interested to see if LARP1 was localized to these sites of RNA fate determination in ovarian cancer cells. After inducing the aggregation of mRNP granules with sodium arsenite treatment, we found LARP1 to be present in both P-bodies and stress granules (Figure 3F, see Supplementary Figure S2D and E for untreated cells). Thus, LARP1 both differentially regulates transcript stability and is present at sites of mRNA fate determination.

### LARP1 is required for cell survival and chemoresistance

**Figure 4.**
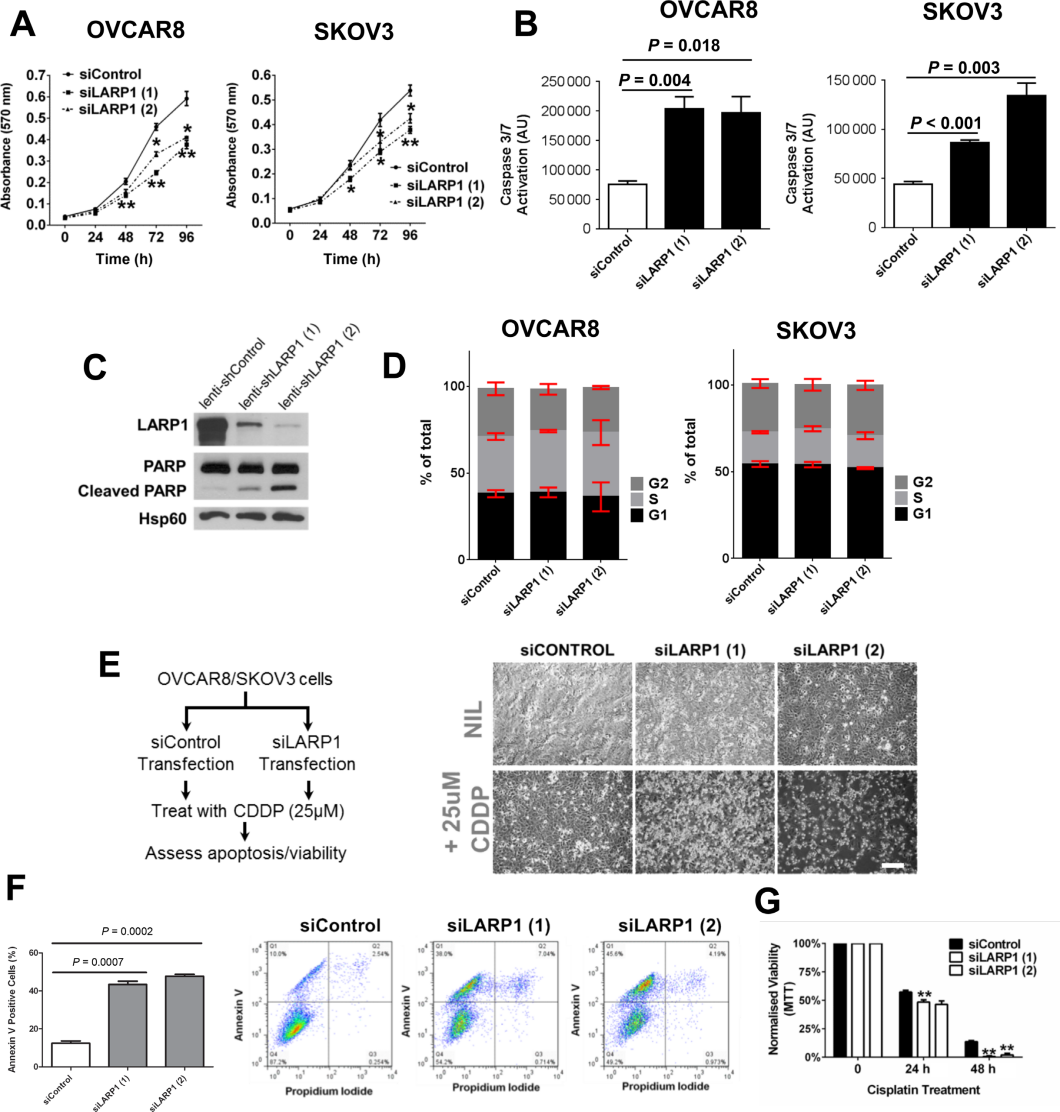
LARP1 knockdown increases basal apoptosis and chemosensitivity. (**A**) Cell viability following transient LARP1 knockdown in OVCAR8 and SKOV3 cells determined by MTT assays. ***P < 0.001, **P < 0.01, *P < 0.05. (**B**) Relative levels of cleaved Caspase 3/7 determined by the CaspaseGlo assay in OVCAR8 and SKOV3 cells 24 h after transient LARP1 knockdown. (**C**) Western blot analysis of cleaved PARP in OVCAR3 cells transduced with lentiviral shLARP1 constructs. (**D**) Cell cycle distribution determined by propidium iodide staining following transient LARP1 knockdown. (**E**) Schematic of cell transfection and cisplatin (CDDP) treatment with representative OVCAR8 cell images in each condition (Scale bar 200 μm). (**F**) Percentage of Annexin V-positive cells following transient LARP1 knockdown and treatment for 24 h with 25 μM cisplatin in platinum-resistant OVCAR8 cells with representative dual-colour flow cytometry plots. (**G**) Normalized cell viability determined by MTT-based assay in OVCAR8 cells following LARP1 knockdown and treatment with 25 μM cisplatin. ***P < 0.001, **P < 0.01, *P < 0.05. Student *t*-test. Minimum of three experimental repeats. Error bars indicate SEM.

We hypothesized that, as LARP1 acted as a post-transcriptional regulator of the apoptosis players BCL2 and BIK, modulation of LARP1 expression in ovarian cancer cells would affect their survival. Indeed, transient LARP1 knockdown decreased cell viability (Figure 4A) and increased apoptosis (Figure 4B and C; Supplementary Figure S3A and B), with no associated change in cell cycle distribution (Figure 4D). Similar trends in cell death were seen in response to hypoxia and nutrient starvation, with LARP1 knockdown increasing apoptosis (Supplementary Figure S3C–E). An important determinant of survival in patients with ovarian cancer is the response of their tumours to chemotherapy, with tumours that are able to evade the pro-apoptotic effects of anti-cancer treatment progressing or recurring more rapidly. As LARP1 knockdown resulted in apoptosis, we investigated whether the protein is required to maintain chemotherapy resistance. Ovarian cancer-derived SKOV3 and OVCAR8 cells are both known to be resistant to platinum-based therapies (44). LARP1 knockdown alone in these lines had minimal effects on cell morphology. As they are resistant to platins, exposure to cis-diamine diplatinum (cisplatin/CDDP) caused minimal change to the microscopic appearance of these cells at 24 hours. However, when combined with LARP1 knockdown, the cells became rounded and detached (Figure 4E). This was associated with up to a 4-fold increase in apoptosis and a significant drop in viability relative to controls (Figure 4F and G and Supplementary Figure S4A–C).

We repeated the experiment with two other chemotherapeutics commonly used to treat EOC, paclitaxel and gemcitabine. With both agents, LARP1 knockdown also led to increased apoptosis and decreased viability compared to drug treatment alone (Supplementary Figure S4D and E). To further evaluate the platinum effect, we obtained isogenic cell lines derived from the same patient before and after the development of platinum resistance (PEO1 and PEO4, respectively). Resistant PEO4 cells have a platinum IC_50_ five times that of their sensitive counterpart (45). Higher LARP1 expression was seen in the platinum-resistant cell line (Supplementary Figure S4F), while knockdown of LARP1 in both lines resulted in greater apoptosis after exposure to cisplatin (Supplementary Figure S4G). These results indicate that LARP1 contributes to apoptosis evasion in ovarian cancer cells and maintains chemotherapy resistance.

### LARP1 is required for tumorigenicity and clonogenicity

**Figure 5.**
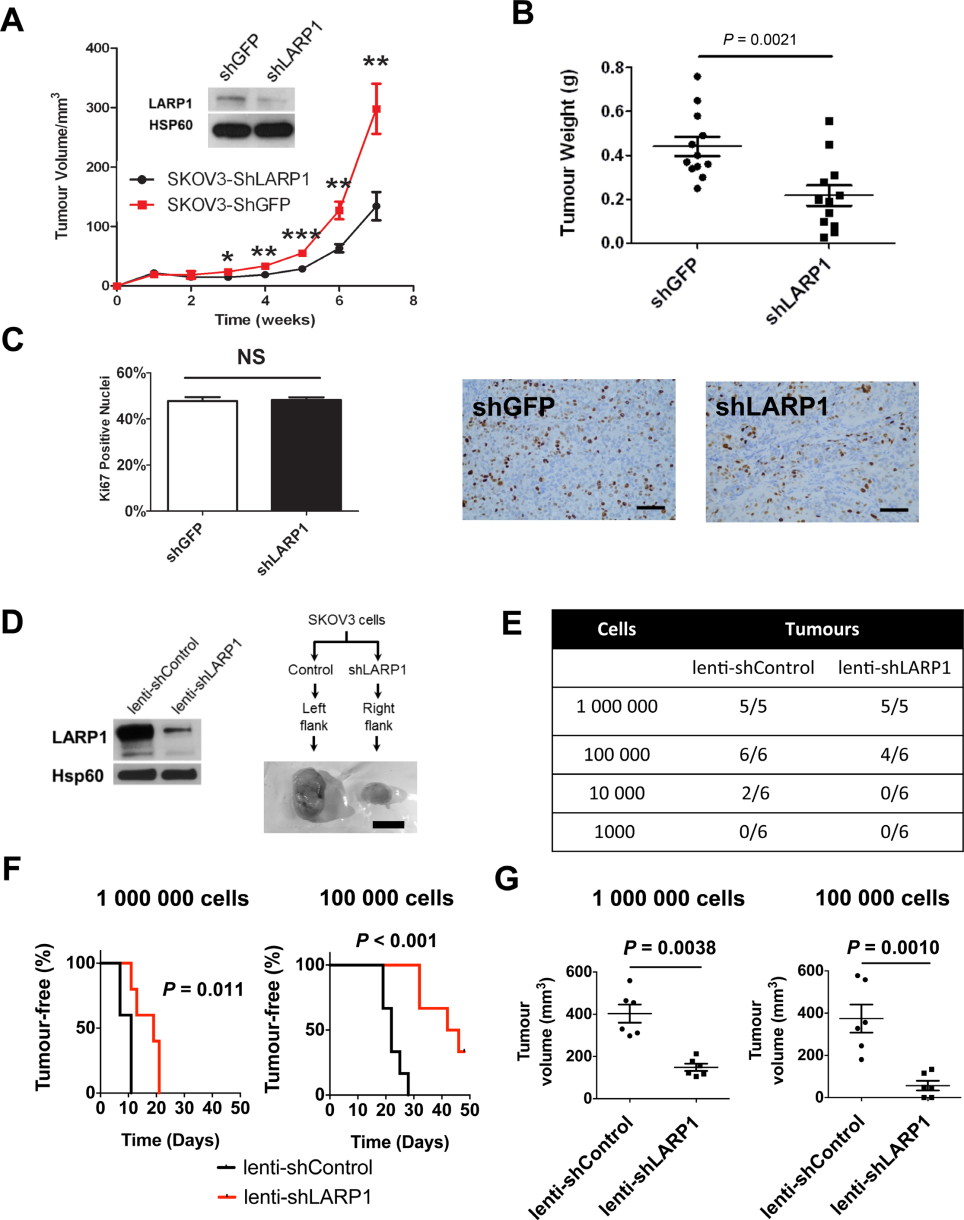
Oncogenic effects of LARP1 *in vivo*. (**A**) SKOV3 control (shGFP) and LARP1 knockdown (shLARP1) cells were injected subcutaneously in SCID-beige mice and tumour volume monitored over time (*inset*, western blot of LARP1 knockdown in implanted cells). (**B**) Final tumour weights at sacrifice. (**C**) Ki67 percentage nuclear positivity of fixed and embedded xenograft tumours analysed by immunohistochemistry and representative examples xenograft tumours stained with anti-Ki67 antibody (scale bar 100 μm). (**D**) Western blot of LARP1 knockdown in SKOV3 stable cell lines using lentiviral transduction, with schematic of cell injection protocol and representative tumours (scale bar 1 cm). (**E**) Limiting dilution assay results from SKOV3 cells injected subcutaneously into NSG mice. (**F**) Kaplan-Meier curves of tumour-free survival for mice receiving 1 × 10^6^ cells (*n* = 5) and 1 × 10^5^ cells (*n* = 6). Log-rank test. (**G**) Final tumour volumes for mice receiving 1 × 10^6^ cells and 1 × 10^5^ cells, respectively. ***P < 0.001, **P < 0.01, *P < 0.05. Student *t*-test. Error bars indicate SEM.

To determine whether LARP1-mediated regulation of cancer cell survival was important for ovarian tumour development, we induced stable knockdown of LARP1 (shLARP1) in SKOV3 cells (Figure 5A, *inset*) and implanted them into SCID-beige mice. Control cells (shGFP) developed measurable tumours with shorter latency compared to shLARP1 cells (median time 22 versus 36 days, *P* = 0.022; Supplementary Figure S5A). Control xenografts also reached significantly larger tumour size (Figure 5A and B). To ascertain whether these differences were due to decreased proliferation in shLARP1 cells, we analysed nuclear positivity of the proliferation marker Ki67 with immunohistochemical (IHC) staining of fixed tumours. As expected, we found no significant difference in proliferation between tumours from each cohort (Figure 5C), suggesting that the effect of LARP1 depletion on cell survival attenuates the tumour-initiating potential of cancer cells. To model this *in vitro*, we carried out clonogenic assays following LARP1 knockdown. We found a significant decrease in colonies formed from ovarian SKOV3 and OVCAR8 cells, and cervical squamous cell carcinoma-derived HeLa cells (Supplementary Figure S5B).

To further investigate whether LARP1 knockdown affected the tumour initiating potential of ovarian cancer cells *in vivo*, we performed a limiting dilution assay, injecting decreasing numbers of SKOV3 cells with stable non-targeting (lenti-shControl) or LARP1-targeting (lenti-shLARP1) short-hairpin expression subcutaneously into NOD-SCID IL2R-gamma^null^ (NSG) mice (Figure 5D). When 10^6^ cells were injected, all mice developed bilateral tumours, though the median latency was considerably greater for tumours with LARP1 knockdown compared to controls (19 versus 11 days, respectively; *P* = 0.011; Figure 5E and F). At 10^5^ cells per injection, measurable tumours were not detected in 2/6 LARP1 knockdown injection sites. Tumour latency was more pronounced, with a median time to tumour development of 22 days in control cells, and 42 days in cells with LARP1-silencing (*P* < 0.001). At 8 weeks following implantation of 10^4^ cells, 2/6 tumours had developed in the control cohort, with no tumours found at sites of shLARP1 cell implantation. No tumours were detected when 10^3^ shControl or shLARP1 cells were injected in either dose cohort (Figure 5E) and tumors took longer to develop in shLARP1 mice compared with controls (Figure 5F). As before, there was a striking difference in tumour volumes between control and LARP1-knockdown tumours (Figure 5G). These data indicate that LARP1 is required for ovarian tumour initiation and progression.

### LARP1 maintains CSC-like populations

**Figure 6.**
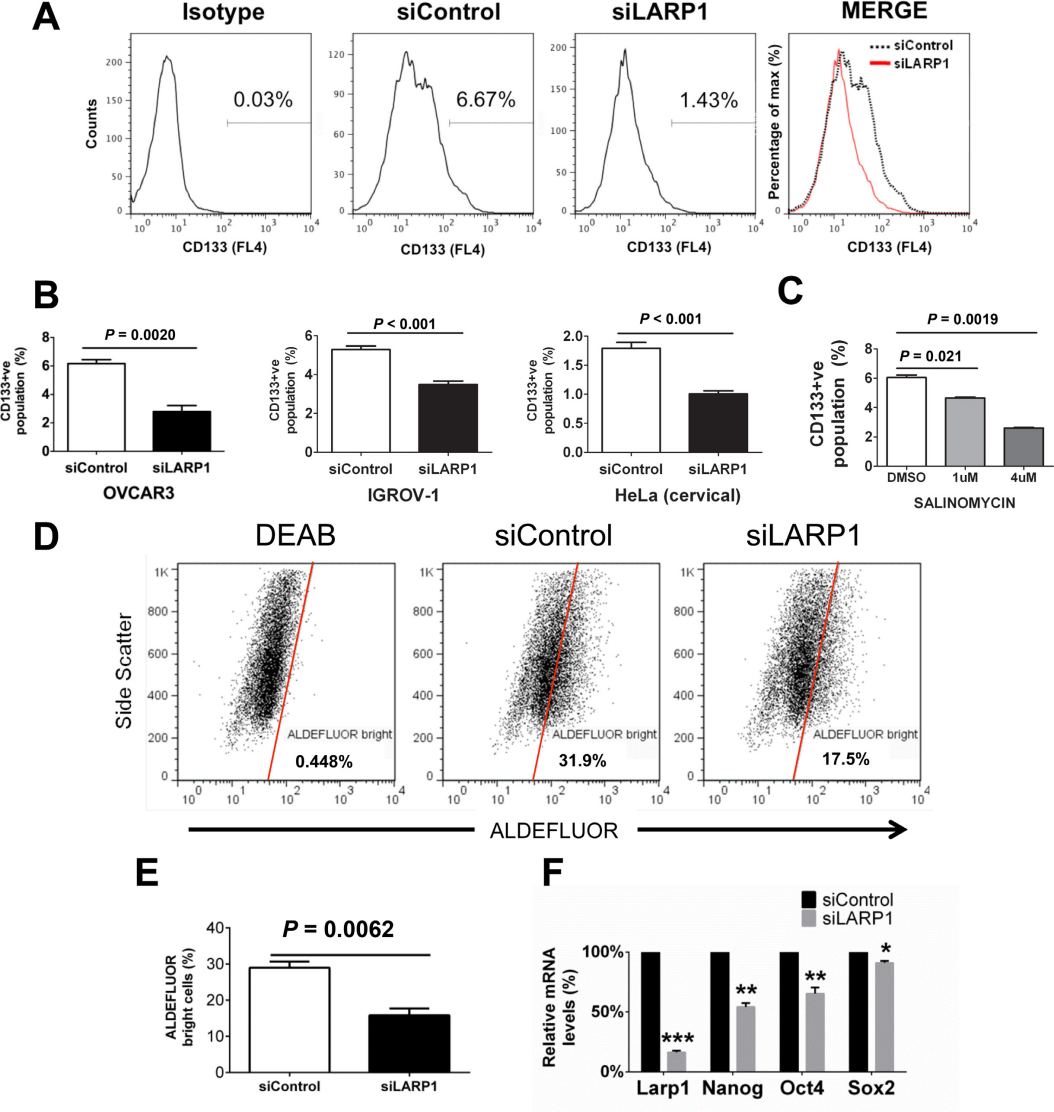
LARP1 is required for the maintenance of populations positive for putative cancer stem cell markers. (**A**) Representative histogram plot of CD133^+^ OVCAR3 cells determined by flow cytometry (a line with <10% CD133-positivity) following LARP1 knockdown. (**B**) Mean CD133^+^ populations determined by flow cytometry following LARP1 knockdown in OVCAR3, IGROV1 and HeLa (cervical carcinoma-derived) cells. (**C**) Percentage of CD133^+^ OVCAR3 cells following treatment with the anti-CSC agent salinomycin. (**D**) Representative flow cytometry plots of ALDEFLUOR^bright^ OVCAR3 cells, with high aldehyde dehydrogenase activity, following LARP1 knockdown, determined using the Aldefluor assay. (**E**) Percentage of ALDEFLUOR^bright^ OVCAR3 cells following LARP1 knockdown determined using the Aldefluor assay. (**F**) Relative mRNA expression of key stem cell-associated transcription factors following LARP1 knockdown (ΔΔ*Ct*). ***P < 0.001, **P < 0.01, *P < 0.05. Student *t*-test. Minimum of three experimental repeats. Error bars indicate SEM.

Enhanced tumorigenicity, clonogenicity and chemotherapy resistance are features often ascribed to cell populations with cancer stem cell (CSC)-like properties (46). Given LARP1 appears to regulate these traits, we hypothesized that the protein may be important in maintaining CSC-like populations. One of the best-characterized markers of CSC-like populations in a range of solid malignancies, including EOC, is CD133 (47). We used the NCI60 panel expression array dataset (37) to identify cell lines with PROM1/CD133 mRNA expression. We found a strong correlation between published mRNA levels and flow cytometry-derived CD133 membrane positivity (Supplementary Figure S6A). Knockdown of LARP1 in OVCAR3 and IGROV-1 lines, and in cervical cancer-derived HeLa cells, resulted in a significant decrease in CD133^+^ cell populations (Figure 6A and B; Supplementary Figure S6B). Having demonstrated that LARP1 targets CSC-like cells, we compared its effects to a positive control, salinomycin, identified as a selective inhibitor of breast cancer stem cell-like populations (48). As expected, salinomycin reduced CD133^+^ populations in OVCAR3 cells, with the highest dose producing an effect equivalent to LARP1 knockdown (Figure 6C).

No single marker has been shown to fully describe intra-tumoral heterogeneity. We assessed the effect of LARP1 knockdown on aldehyde dehydrogenase (ALDH) activity, another commonly used CSC marker. LARP1 knockdown resulted in a decrease in ALDH activity (Figure 6D and E). High expression of several key embryonic stem cell-related transcription factors have been associated with enhanced CSC-like traits, including *SOX2, OCT4* and *NANOG* (49). Following LARP1 knockdown, we found reduction in expression of all three genes, with the most pronounced effect on *NANOG* (Figure 6F). These results show that LARP1 promotes several characteristics associated with CSC-like cells, namely clonogenicity, tumorigencitiy, chemotherapy resistance, expression of stem cell-associated genes and maintenance of CSC marker-positive populations.

### LARP1 enhances cell survival by promoting BCL2 expression

**Figure 7.**
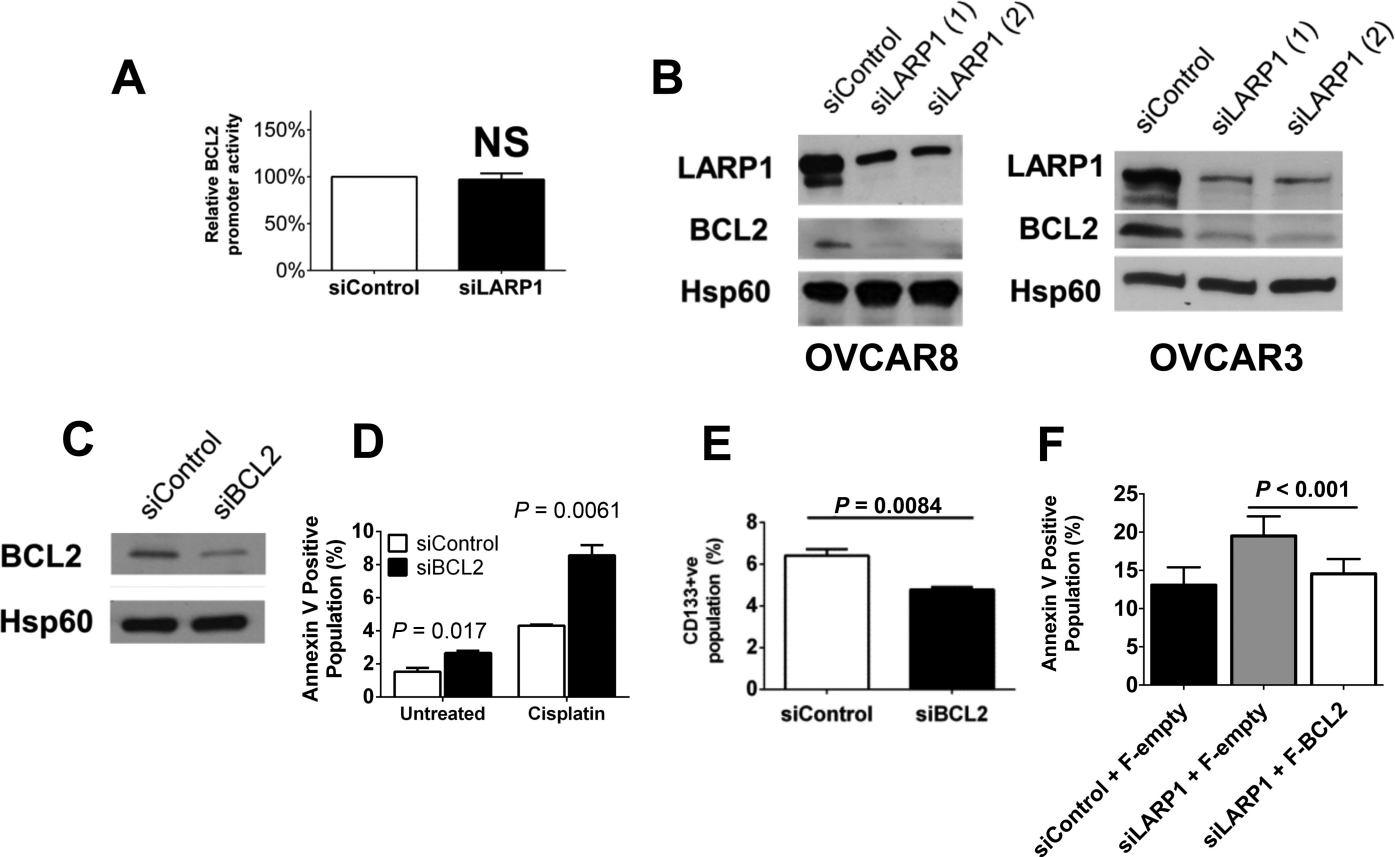
BCL2 can rescue the LARP1 apoptotic phenotype. (**A**) BCL2 promoter activity following LARP1 knockdown (*firefly* luciferase mRNA normalized to *renilla* luciferase mRNA control). (**B**) Western blot analysis of BCL2 protein levels following LARP1 knockdown. (**C**) Western blot analysis of BCL2 protein levels following BCL2 knockdown. (**D**) Percentage of Annexin V-positive OVCAR8 cells, determined by flow cytometry, following transient BCL2 knockdown, with and without co-treatment with cisplatin (25 μM). (**E**) Percentage of CD133-posititve OVCAR3 cells, determined by flow cytometry, following transient knockdown of BCL2. (**F**) Percentage of Annexin V-positive OVCAR8 cells, determined by flow cytometry, following LARP1 knockdown or transfection with control siRNA and treatment with cisplatin (25 μM), with co-transfection of a Flag-tagged control (F-empty) or BCL2-overexpression (F-BCL2) construct. Minimum of three experimental repeats. Student *t*-test. Error bars indicate SEM.

Our data demonstrate that BCL2 is an important promoter of chemotherapy resistance and appears to be required for CSC survival (39,40). We next investigated the effect of LARP1 knockdown on *BCL2* promoter activity. No change was observed following LARP1 knockdown (Figure 7A), confirming that the altered *BCL2* transcript abundance expression seen on reduced LARP1 expression (Figure 1C) and resultant change in BCL2 protein levels (Figure 7B) were due to alterations in mRNA stability alone. To determine if the changes in BCL2 expression on LARP1 knockdown were sufficient to explain the observed phenotypes, we used *BCL2*-targeting siRNA to reduce expression (Figure 7C). As expected, decreased expression of BCL2 resulted in increased apoptosis in response to platinum treatment (Figure 7D), and also reductions in CD133^+^ CSC-like populations (Figure 7E). We next assessed the ability of BCL2 overexpression to rescue the effects of LARP1 depletion. Indeed, following knockdown of LARP1 and treatment with cisplatin, transfection with a FLAG-tagged BCL2 overexpression construct resulted in a significant decrease in apoptosis when compared to control plasmid-transfected cells (Figure 7F). These data indicate LARP1 promotes *BCL2* mRNA stability and protein expression, without which effect cells demonstrate increased apoptosis and decreased chemotherapy resistance.

### LARP1 is highly expressed in ovarian cancer and predicts prognosis

**Figure 8.**
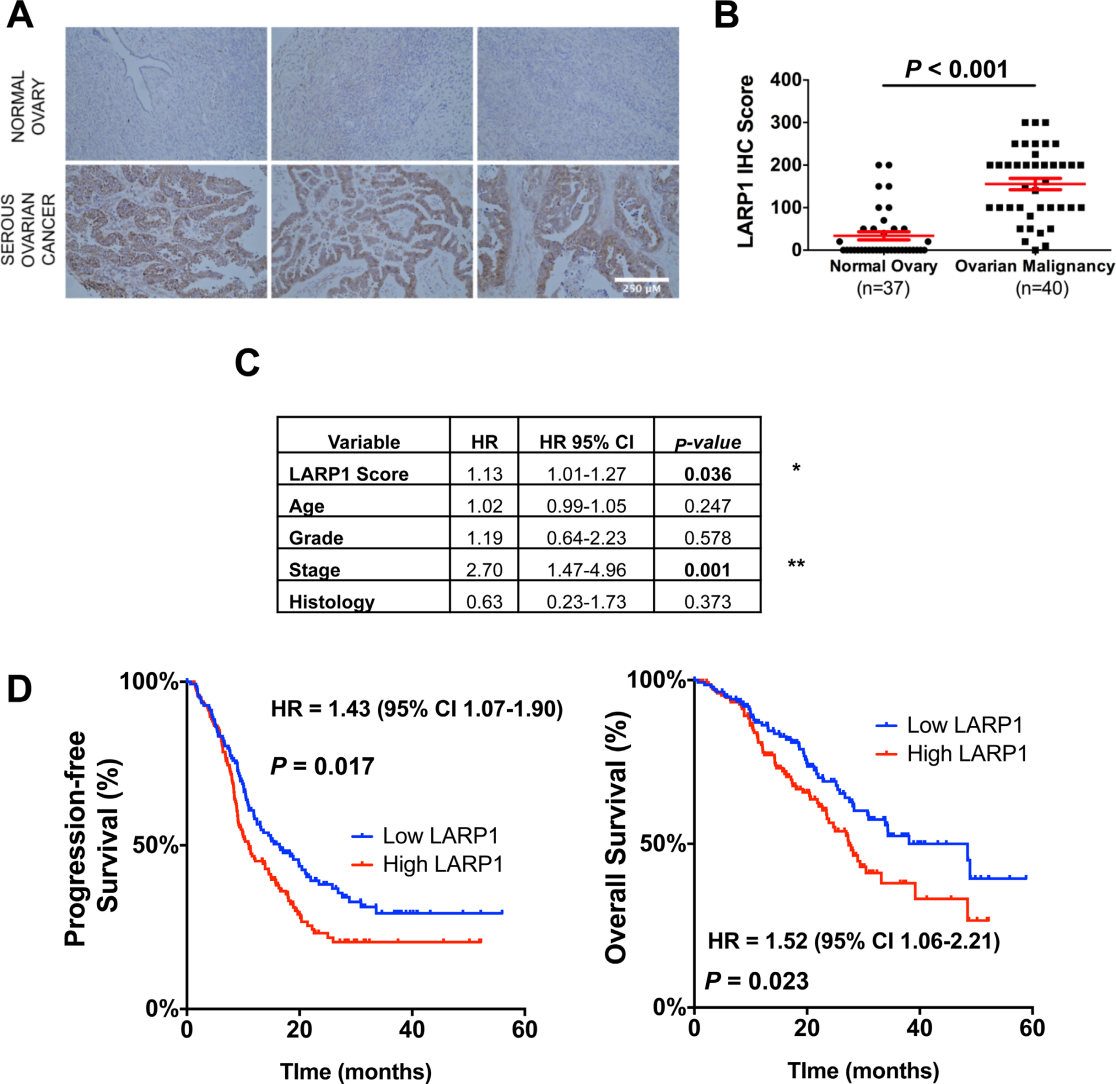
LARP1 is highly expressed in ovarian cancers and predicts poor prognosis. (**A**) Representative TMA cores of normal ovarian tissue and EOC samples, stained with anti-LARP1 antibody (scale bar 250 μm). (**B**) LARP1 immunohistochemical scoring in healthy ovaries and ovarian cancers. (**C**) Multivariate cox-regression analysis of overall survival in 67 cases of ovarian cancer. Hazard ratios for LARP1 score, as determined by IHC analysis of sectioned tumours, given for a 10-point change in score. (**D**) A TMA of pre-treatment ovarian tumour tissue from 281 participants in the SCOTROC4 study was stained with anti-LARP1 antibody, and scored independently. Kaplan-Meier analysis of progression-free survival (PFS) and overall survival (OS) is shown, with patients stratified by LARP1 score (Median PFS 16.5 versus 11.0 months and OS 48.5 versus 27.4 months in LARP1 low and high tumours, respectively).

Having demonstrated LARP1 promotes ovarian cancer cell survival, we investigated LARP1 expression in ovarian malignancies. First, we extracted data from three independent expression array studies representing 735 patient samples (32–34). LARP1 mRNA levels were significantly upregulated in serous EOC, the most common epithelial subtype (50), compared to non-malignant ovarian tissue in all three studies (Supplementary Figure S7A). Similar trends were observed comparing LARP1 mRNA expression in serous EOC to normal ovarian surface, and fallopian tube, epithelium (51,52) (*P* = 0.0072 and 0.022 respectively; Supplementary Figure S7B and C). Using IHC analysis of ovarian TMAs, we found significantly higher expression of LARP1 protein in ovarian cancer samples compared to normal ovarian tissue (*P* < 0.001; Figure 8A and B). Similar results were obtained comparing serous ovarian cancers with benign ovarian tumours (*P* = 0.021; Supplementary Figure S7D), and benign and malignant mucinous ovarian tumours, rarer histological EOC subtypes (*P* = 0.033; Supplementary Figure S7E).

Analysis of overall survival, using matched mRNA expression and patient outcome data obtained from The Cancer Genome Atlas (TCGA) project (32), revealed that patients with the highest LARP1 expression had significantly worse outcomes, with a 29% increased risk of death at any time (*n* = 566, HR 1.29, 95% CI 1.01–1.65, *P* = 0.042). We then assessed the effect of LARP1 expression on PFS in ovarian cancer. In an analysis of 1171 patient samples, those with low LARP1 expression had significantly better PFS than those with high LARP1 expression (HR 1.31, 95% CI 1.10–1.54, *P* = 0.0018; Supplementary Figure S7F). Interestingly, this finding was not limited to ovarian cancer; by analysing overall survival in 1115 breast cancer patients, we found that high LARP1 expression was also predictive of poor outcome (HR = 1.53, *P* < 0.001, 95% CI 1.2–1.96; Supplementary Figure S7G), suggesting an oncogenic role for LARP1 in other tumour types.

In an analysis of a TMA of 67 ovarian cancer cases collected at initial debulking surgery, we showed that only LARP1 expression and cancer stage were significant independent predictors of poor overall survival in patients with ovarian malignancy (LARP1 HR = 1.13, 95% CI 1.01–1.27, *P* = 0.036; Figure 8C). To study this survival effect in greater depth we obtained patient samples collected as part of Trans-SCOTROC, a translational substudy within SCOTROC IV (53). This was a multi-centre, randomized trial of flat-dosed (AUC 6) versus intrapatient dose-escalated (median of AUC 7.2) single-agent carboplatin as first-line chemotherapy in 964 women with stage IC-IV epithelial ovarian, fallopian tube or serous peritoneal cancer. It was conducted between 2004 and 2009, before futility analysis confirmed a lack of superiority of the intrapatient dose-escalated arm. A TMA was created using tumour samples collected from 281 participants at primary debulking surgery. This was stained using anti-LARP1 antibody and scored blind by two independent histopathologists (inter-rater kappa coefficient of 0.7) with LARP1 levels scored as ‘low’ or ‘high’. LARP1 expression was low in 139 patients and high in 142. When patients were stratified according to LARP1 expression, those with high LARP1 expression had a significantly worse PFS (median 11.0 versus 16.5 months, *P* = 0.019) and overall survival (27.4 versus 48.5 months, *P* = 0.028) (Figure 8D).

**Figure 9.**
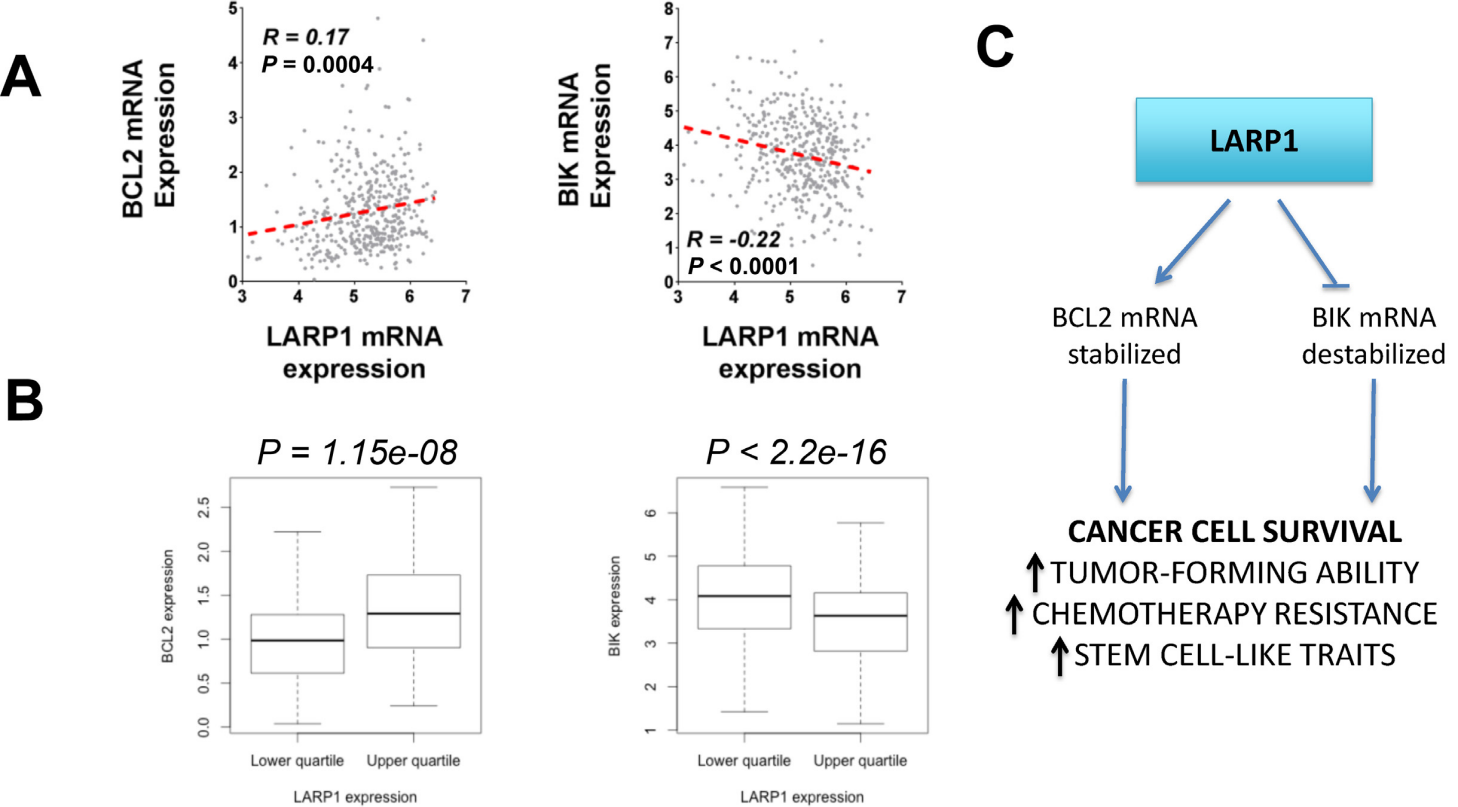
LARP1 expression in ovarian tumors correlates with BIK and BCL2 expression. (**A**) Correlation of mRNA expression (Log_2_RPKM) in ovarian tumors (*n* = 412) between LARP1 and BCL2 or BIK (Pearson R). Data from TCGA Ovarian RNAseq cohort (tcga-data.nci.nih.gov). (**B**) Comparison of upper and lower quartiles of LARP1 expression (Log_2_RPKM, *n* = 103 in each), by BCL2 or BIK expression (Wilcoxon test). Data as before. (**C**) A summary of the role of LARP1 in the ovarian cancer cell. As a component of mRNP complexes containing transcripts of survival-associated genes, LARP1 acts to simultaneously stabilize anti-apoptotic mRNAs, whilst destabilizing pro-apoptotic transcripts. The net effect is to promote apoptosis evasion, enhancing tumorigenicity, chemoresistance and cancer stem cell (CSC)-like traits.

To determine whether LARP1 could be regulating the stability of *BCL2* and *BIK* transcripts in patient tumours, we evaluated trends in *LARP1, BCL2* and *BIK* transcript abundance in the TCGA ovarian cancer dataset. As expected, *LARP1* and *BCL2* mRNA levels showed a significant positive correlation, while *LARP1* and *BIK* expression were negatively correlated (Figure 9A and B). Together with our *in vitro* and *in vivo* data, this suggests a model whereby LARP1 differentially regulates the mRNA stability of pro- and anti-apoptotic transcripts in malignant ovarian tumours to promote cell survival (Figure 9C).

**Figure 10.**
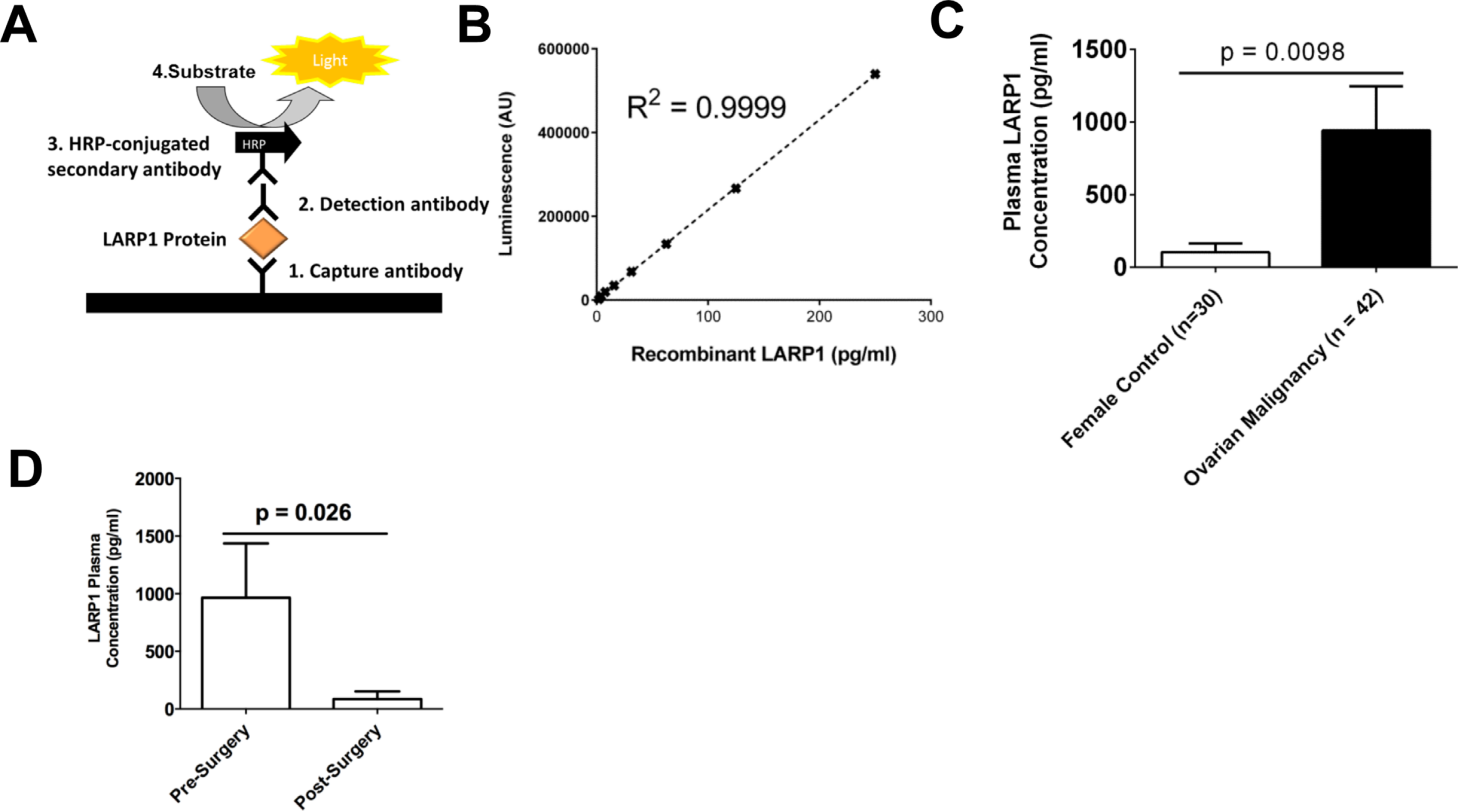
LARP1 protein is detectable in patient blood plasma. (**A**) A schematic of the LARP1 sandwich enzyme-linked immunosorbent assay (ELISA). (**B**) The LARP1 ELISA standard curve. (**C**) Plasma LARP1 concentration in healthy female controls and patients with primary ovarian malignancies sampled prior to primary surgery. (**D**) Plasma LARP1 concentration for 19 patients with underlying ovarian malignancy sampled before and after primary surgery. Students *t*-test. Error bars indicate SEM.

As LARP1 is highly expressed in ovarian tumours, we hypothesized that patients with underlying malignancy may have higher levels of circulating protein. To investigate this we developed a sandwich enzyme-linked immunosorbent assay (ELISA) to accurately quantify LARP1 protein in human plasma (Figure 10A and B). We found significantly higher levels of circulating LARP1 protein in plasma from women with underlying ovarian malignancy, than healthy controls (Figure 10C). Analysing a subset of patients for which there were paired plasma samples taken before and after first-line surgical debulking, we found all patients exhibited a drop in circulating LARP1 protein (Figure 10D). Though larger studies are required, these data suggest potential as a circulating, as well as intratumoral, biomarker.

## DISCUSSION

LARP1 promotes both the stabilization (13,15) and destabilization (14) of selected messenger RNA transcripts. A similar role has been ascribed to other LARPs including LARP4b (54), suggesting a conserved function within the LARP family. Here, we investigated the mRNA stability-regulating role of LARP1 in EOC. We utilized transcriptomic analysis following LARP1 knockdown, cross-referenced against the LARP1 mRNA interactome, to identify potential LARP1 mRNA targets. We demonstrated that genes with altered transcriptomic abundance on knockdown were more likely to be represented in the interactome. These transcripts were enriched for functions linked to cell death and survival, and included *BIK* and *BCL2*. Previous studies of LARP1a in *Arabidopsis* suggest that LARP1 may have a net stabilizing effect on its target transcripts (14). However, we found that an equal proportion of mRNAs showed increased and decreased abundance following LARP1 knockdown. We also found that LARP1 can simultaneously differentially regulate transcript fate, stabilizing transcripts of the anti-apoptotic gene *BCL2*, whilst destabilizing pro-apoptotic *BIK* mRNAs. Although representing opposing effects on RNA stability, the anticipated net consequence of increased LARP1 is to enhance cancer cell survival (Figure 9C). Indeed, we found that LARP1 is required for apoptosis evasion and to ensure cell survival following exposure to chemotherapy in platinum-resistant ovarian cancer cells. Laboratory culture conditions provide cells with a permissive environment for optimal growth, whilst implantation into host animals presents potential additional apoptotic triggers, such as decreased oxygen and nutrient availability. As well as increased apoptosis under hypoxic and nutrient-depleted conditions, LARP1 knockdown *in vivo* was associated with attenuated clonogenicity and tumorigenicity. We established that LARP1 exerts this pro-survival effect, at least in part, by post-transcriptionally promoting the expression of BCL2, a well-characterized oncogenic anti-apoptotic protein and a promoter of cancer cell survival (55). Upregulation of BCL2 is a negative prognostic factor in ovarian cancer (56) and induces platinum resistance (39). In ovarian cancer cells, we showed that knockdown of BCL2 causes apoptosis and enhanced chemosensitivity, while overexpression of BCL2 partially rescues the effects of LARP1 depletion. On LARP1 knockdown, we also observed increased mRNA levels of pro-apoptotic genes, such as *BIK* and *DAPK2*, and reduced expression of anti-apoptotic genes, including *ERBB3* and *AKT3*. Given these changes, the apoptosis observed following LARP1 knockdown is likely due to alterations in expression of multiple LARP1 targets.

Using mouse models of ovarian malignancy, it has been proposed that transformed stem cells may be the origin of at least some types of EOC (22). Flow cytometry markers, such as CD133 membrane expression and aldehyde dehydrogenase activity, can identify sub-populations of ovarian cancer cells demonstrating CSC-like characteristics (47). As well as a role in regulating key CSC-associated traits such as clonogencity, tumour-initiating capability and chemotherapy resistance, LARP1 is required to maintain CD133^+^ and Aldefluor^bright^ putative CSC-like populations. LARP1 knockdown also leads to reduced expression of stem cell-related transcription factors. Interestingly, LARP1 was one of the most strongly downregulated genes in non-CSC-like CD133^−^ populations (57) in a study investigating global differences in gene expression between CD133^+^ and CD133^−^ cells derived from a patient with progressive glioblastoma multiforme. LARP1 has also been shown to bind mRNA in embryonic stem cells, and expression of LARP1 falls during cell differentiation (58). This suggests LARP1 may be an important factor in the maintenance of stem cell-like traits. Expression of BCL2 is elevated in CSC-like populations and targeted BCL2 inhibitors selectively kill leukemic cancer stem cell populations (40). In ovarian cancer cells, we showed that knockdown of BCL2 also reduces CSC-like populations, suggesting that the LARP1-mediated anti-CSC phenotype may also be mediated via BCL2 expression.

Post-transcriptional regulation of mRNA expression is coordinated by elements within the 5′ UTR of transcripts that can affect translation efficiency, and 3′ UTR *cis*-acting features that determine message stability (42). LARP1 interacts with poly(A)-binding protein (PABP) (27) and has been previously been identified as part of a 3′ UTR-associated mRNP complex (13). We show here that the 3′ UTR is critical to the stability-regulating function of LARP1 and, in the case of *BCL2*, the DM15 region of LARP1 is the site with which it binds the 3′ UTR of its target mRNA. One of the best characterized 3′ UTR stability-determining features are AU-rich elements (AREs), recognized by RBPs that induce degradation, whilst proteins that compete for this interaction can promote stability (42). *BCL2* has a well-characterized ARE-rich region (*BCL2-ARE*) that determines its mRNA stability (29); however the LARP1 stability-regulating effect acts independently of this region.

Here, we report for the first time that LARP1 expression is upregulated in ovarian malignancies, with high intratumoral levels associated with adverse prognosis. LARP1 has previously been identified as a predictor of poor prognosis in hepatocellular (18) and lung (15) cancers, indicating it may be important in driving malignant progression across multiple tumour types. Supporting a clinically meaningful role for LARP1 in the post-transcriptional regulation of BCL2 and possibly also BIK expression in ovarian malignancies, expression of *LARP1* positively correlates with *BCL2*, but is negatively correlated with *BIK* expression in a study of over 400 ovarian tumours. Like LARP1, the RBP HuR has previously been shown to have a dual effect on mRNA stability, stabilizing oncogenic transcripts such as VEGF (59), whilst destabilizing transcripts encoding the tumour suppressor p16INK4 (60). Similarly, HuR has also been associated with chemotherapy resistance (61), suggesting differential regulation of stability by oncogenic RBPs is not an uncommon event.

There is increasing evidence linking the LARP protein family to cancer (11). By differentially regulating the stability of pro- and anti-apoptotic mRNA transcripts, LARP1 acts as a post-transcriptional promoter of apoptosis evasion in ovarian cancer cells. Our discovery of a correlation between circulating levels of LARP1 and tumour burden in ovarian cancer patients suggests that plasma LARP1 levels could prove a useful non-invasive means of measuring tumour LARP1 expression, which we intend to prospectively validate. Our study suggests that, through its post-transcriptional control of chemosensitivity, LARP1 is a potential future therapeutic target for the reversal of chemotherapy-resistant disease and may have utility as an accompanying biomarker.

## SUPPLEMENTARY DATA

Supplementary Data are available at NAR Online.

SUPPLEMENTARY DATA
