# Supplementary material for: The RNA-binding protein LARP1 is a post-transcriptional regulator of survival and tumorigenesis in ovarian cancer
Source: Nucleic Acids Res. 2015 Dec 29;44(3):1227–46. doi: 10.1093/nar/gkv1515 (PMC4756840; doi:10.1093/nar/gkv1515)
Supplement: SUPPLEMENTARY DATA [file supp_44_3_1227__index.html]

The RNA-binding protein LARP1 is a post-transcriptional regulator of survival and tumorigenesis in ovarian cancer — The RNA-binding protein LARP1 is a post-transcriptional regulator of survival and tumorigenesis in ovarian cancer — SUPPLEMENTARY DATA 

# The RNA-binding protein LARP1 is a post-transcriptional regulator of survival and tumorigenesis in ovarian cancer

## SUPPLEMENTARY DATA

- SUPPLEMENTARY DATA
- SUPPLEMENTARY DATA
