# Supplementary material for: The RNA-binding protein LARP1 is a post-transcriptional regulator of survival and tumorigenesis in ovarian cancer
Source: Nucleic Acids Res. 2015 Dec 29;44(3):1227–46. doi: 10.1093/nar/gkv1515 (PMC4756840; doi:10.1093/nar/gkv1515)
Supplement: SUPPLEMENTARY DATA [file supp_gkv1515_nar-02000-2015-File003.docx]

# SUPPLEMENTARY FIGURE LEGENDS

Supplementary Figure 1. Knockdown of LARP1 alters the cancer cell transcriptome.

(**A**) Transcript abundance of LARP family members in the RNA-seq analysis of control samples.

(**B**) Disease enrichment with Ingenuity Pathway Analysis (IPA) of genes differentially expressed following LARP1 knockdown (-log[BH-corrected p-value] shown, red dashed line indicates p = 0.05).

(**C**) Molecular ontology enrichment with Ingenuity Pathway Analysis (IPA) of genes differentially expressed following LARP1 knockdown (-log[BH-corrected p-value] shown, red dashed line indicates p = 0.05).

**(D)** SKOV3 and OVCAR8 cells were co-transfected with *Renilla* luciferase 5’UTR reporter constructs for BIK and a control *Firefly* luciferase control vector. *Renilla* luciferase activity was determined following LARP1 siRNA and normalized to *Firefly* luciferase activity.

Supplementary Figure 2. LARP1 EMSAs and LARP1 subcellular localization.

(**A, B**) Electrophoretic mobility shift assays of LARP1 and the 3'UTRs of BIK and BCL2.  Top, diagrams showing locations of constructs to scale. Bottom, gel shifts with indicated constructs. Binding constants are shown (n=3). (**C)** control constructs: OAZ1 3'UTR, BIK coding sequence and BIK 5’UTR (**D**) Representative immunofluorescence image of SKOV3 cells stained with anti-LARP1 antibody (green) and anti-PABP antibody (red) without arsenite treatment. (**E**) Representative immunofluorescence image of SKOV3 cells stained with anti-LARP1 antibody (green) and anti-DCP1A antibody (red) without arsenite treatment.

Supplementary Figure 3. LARP1 knockdown increases apoptosis.

(**A**) Representative histogram plot of Annexin V-positive cells determined by flow cytometry in OVCAR8 cells 24 hours after transfection with LARP1-targeting siRNA

(**B**) Percentage of Annexin V-positive cells at 24 hours following transient LARP1 knockdown in OVCAR8 and SKOV3 cells.

(**C**) Apoptosis determined by the CaspaseGlo assay in response to hypoxia in OVCAR8 and SKOV3 cells following LARP1 knockdown. Cells were transferred to a hypoxic environment and transient LARP1 knockdown was performed at T=0 and T=24hrs, with apoptosis recorded at each timepoint.

(**D**) Apoptosis in SKOV3 cells following LARP1 knockdown and exposure to FCS-reduced (0.1%) conditions. Following LARP1 knockdown, cells were transferred to serum-reduced conditions and apoptosis was recorded at each time point (data normalised to apoptosis immediately after completion of LARP1 knockdown).

(**E**) Apoptosis in SKOV3 cells following LARP1 knockdown and exposure to L-glutamine-depleted conditions. Following LARP1 knockdown, cells were transferred to L-glutamine-depleted conditions and apoptosis was recorded at each time point (data normalised to apoptosis immediately after completion of LARP1 knockdown). ***P < 0.001, **P < 0.01, *P < 0.05. Student t-test. Minimum of three experimental repeats. Error bars indicate SEM.

Supplementary Figure 4. LARP1 protein is required for chemoresistance.

(**A**) Percentage of Annexin V-positive cells following transient LARP1 knockdown and treatment for 24 hours with 25μM cisplatin in platinum-resistant SKOV3 cells.

(**B**) Representative dual-colour flow cytometry plots of Annexin V-FITC- and PI-stained SKOV3 cells transfected with LARP1-targeting siRNA and treated with with 25μM cisplatin.

(**C**) Normalised cell viability determined by MTT-based assay in SKOV3 cells following LARP1 knockdown and treatment with 25μM cisplatin.

(**D**) Apoptosis measured by cleaved caspase-3/7 in SKOV3 cells following 24 hours exposure to paclitaxel (1µM) or gemcitabine (2µM).

(**E**) Cell viability determined by MTT assay in SKOV3 cells following exposure to paclitaxel (1µM) or gemcitabine (2µM).

(**F**) Relative LARP1 mRNA expression in PEO1 and PEO4 cells determined by RT-qPCR

(**G**) Percentage of Annexin V-positive platinum-sensitive PEO1 cells following transient LARP1 knockdown and treatment with 10μM cisplatin, and platinum-resistant PEO4 cells following transient LARP1 knockdown and treatment with 25μM cisplatin. ****P* < 0.001, ***P* < 0.01, **P* < 0.05. Student t-test. Minimum of three experimental repeats. Error bars represent SEM.

Supplementary Figure 5. LARP1 promotes tumorigenicity and clonogenicity.

(**A**) Kaplan-Meier curves of tumor-free survival. Log-rank test.

(**B**) Clonogenic assays performed in three cell lines. Representative 10cm plates shown (scale bar 2cm). ****P* < 0.001, ***P* < 0.01, **P* < 0.05. Student t-test. Minimum of three experimental repeats. Error bars indicate SEM.

Supplementary Figure 6. LARP1 is required for the maintenance of CSC-marker positive populations.

(**A**) Experimentally-derived CD133-positive population frequency in ovarian cancer cell lines compared with published CD133 relative mRNA abundance as determined by expression array analysis of the NCI60 cell panel(22).

(**B**) Western blot analysis of LARP1 knockdown in OVCAR3, IGROV1 and HeLa cells.

Supplementary Figure 7. LARP1 is highly expressed in ovarian cancers

(**A**) LARP1 mRNA fold change in serous ovarian cancers compared to control tissue in 3 independent datasets (TCGA (31), Hendrix et al. (33), Bonome et al. (32)). Student t-test.

(**B**) Relative LARP1 mRNA abundance in ovarian surface epithelium (OSE, n=12) and microdissected serous papillary ovarian cancer (SPOC, n=12). Dataset GSE14407, Bowen et al. (34).

(**C**) Relative LARP1 mRNA abundance in fallopian tube epithelium (OSE, n=12) and high-grade serous ovarian cancer (HGOC, n=13). Dataset GSE10971, Tone et al. (35).

(**D**) LARP1 score determined by IHC analysis in benign ovarian tumors (leiomyoma, teratoma and cystadenofibroma) and serous ovarian cancers.

(**E**) LARP1 score in mucinous ovarian tumors determined by IHC analysis, together with representative images (10x magnification). Student t-test. Error bars indicate SEM.

(**F**) Kaplan-Meier analysis of PFS in ovarian cancer patients, separated by LARP1 expression (n=1,171). Data from kmplot.com (48).

(**G**) Kaplan-Meier analysis of overall survival in breast cancer patients, separated by LARP1 expression (n=1,115). Data from kmplot.com (49).
