# Supplementary material for: The RNA-binding protein LARP1 is a post-transcriptional regulator of survival and tumorigenesis in ovarian cancer
Source: Nucleic Acids Res. 2015 Dec 29;44(3):1227–46. doi: 10.1093/nar/gkv1515 (PMC4756840; doi:10.1093/nar/gkv1515)
Supplement: SUPPLEMENTARY DATA [file supp_gkv1515_nar-02000-2015-File004.pptx]

## Slide 1
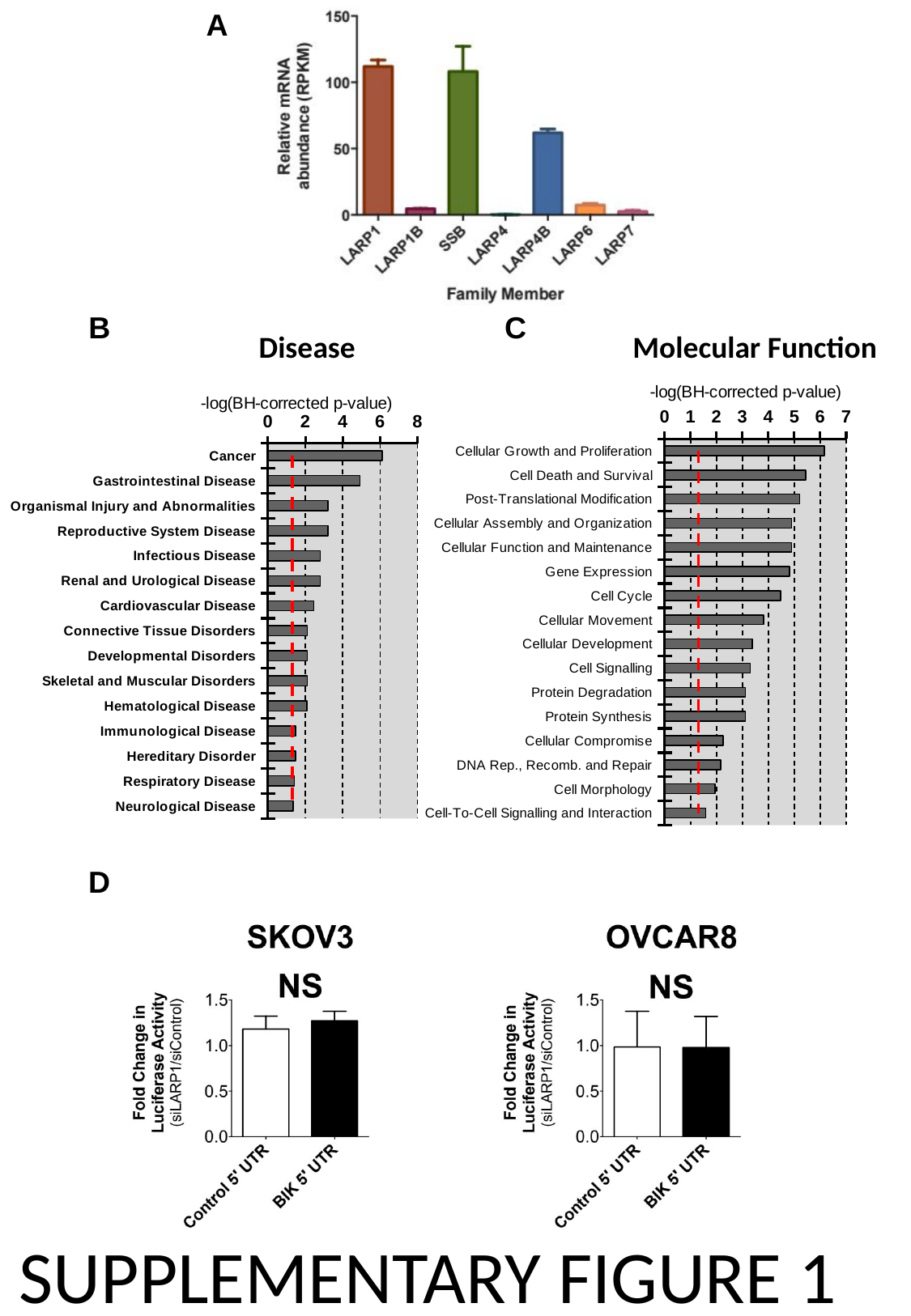

A
C
B
Disease
### Chart: -log(BH-corrected p-value)
| Category | -log(BH-corrected p-value) | |
|---|---|---|
| Cancer | 6.120904120499874 | 1.301029995663981 |
| Gastrointestinal Disease | 4.928117992693874 | 1.301029995663981 |
| Organismal Injury and Abnormalities | 3.206209615309179 | 1.301029995663981 |
| Reproductive System Disease | 3.206209615309179 | 1.301029995663981 |
| Infectious Disease | 2.801342913045576 | 1.301029995663981 |
| Renal and Urological Disease | 2.801342913045576 | 1.301029995663981 |
| Cardiovascular Disease | 2.447331783887807 | 1.301029995663981 |
| Connective Tissue Disorders | 2.096910013008054 | 1.301029995663981 |
| Developmental Disorders | 2.096910013008054 | 1.301029995663981 |
| Skeletal and Muscular Disorders | 2.096910013008054 | 1.301029995663981 |
| Hematological Disease | 2.077793722560984 | 1.301029995663981 |
| Immunological Disease | 1.473660722610156 | 1.301029995663981 |
| Hereditary Disorder | 1.471083299722345 | 1.301029995663981 |
| Respiratory Disease | 1.405607449624573 | 1.301029995663981 |
| Neurological Disease | 1.357535479757878 | 1.301029995663981 |Molecular Function
### Chart: -log(BH-corrected p-value)
| Category | -log(BH-corrected p-value) | |
|---|---|---|
| Cellular Growth and Proliferation | 6.165579296318401 | 1.301029995663981 |
| Cell Death and Survival | 5.44129142946684 | 1.301029995663981 |
| Post-Translational Modification | 5.207608310501746 | 1.301029995663981 |
| Cellular Assembly and Organization | 4.892790030352131 | 1.301029995663981 |
| Cellular Function and Maintenance | 4.892790030352131 | 1.301029995663981 |
| Gene Expression | 4.80966830182971 | 1.301029995663981 |
| Cell Cycle | 4.467245621007502 | 1.301029995663981 |
| Cellular Movement | 3.815308569182401 | 1.301029995663981 |
| Cellular Development | 3.375717904164331 | 1.301029995663981 |
| Cell Signalling | 3.302770657240282 | 1.301029995663981 |
| Protein Degradation | 3.114073660198568 | 1.301029995663981 |
| Protein Synthesis | 3.114073660198568 | 1.301029995663981 |
| Cellular Compromise | 2.262012673666569 | 1.301029995663981 |
| DNA Rep., Recomb. and Repair | 2.166215625343519 | 1.301029995663981 |
| Cell Morphology | 1.950781977329818 | 1.301029995663981 |
| Cell-To-Cell Signalling and Interaction | 1.578396073130169 | 1.301029995663981 |D
SUPPLEMENTARY FIGURE 1

## Slide 2
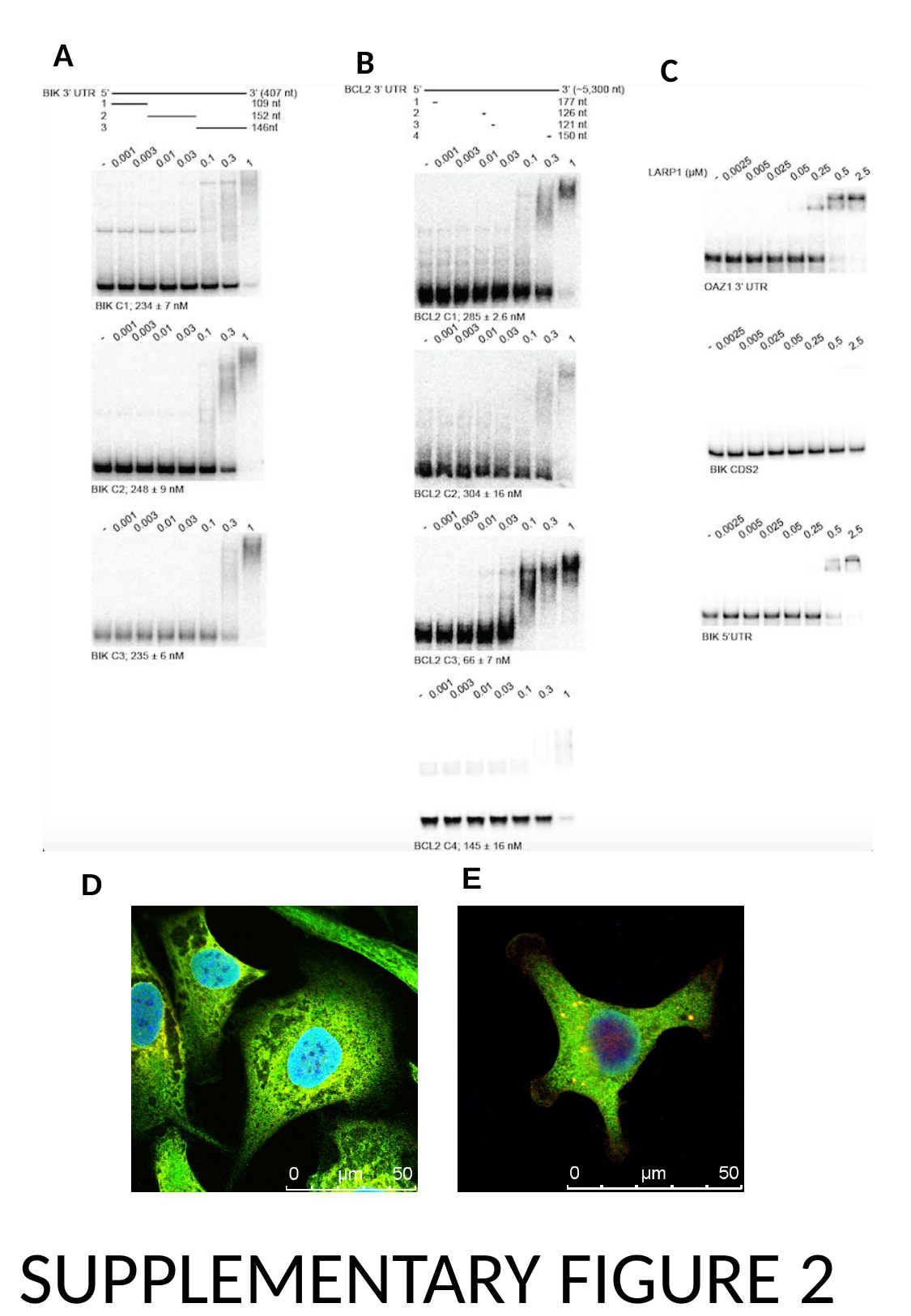

A
B
C
E
D
SUPPLEMENTARY FIGURE 2

## Slide 3
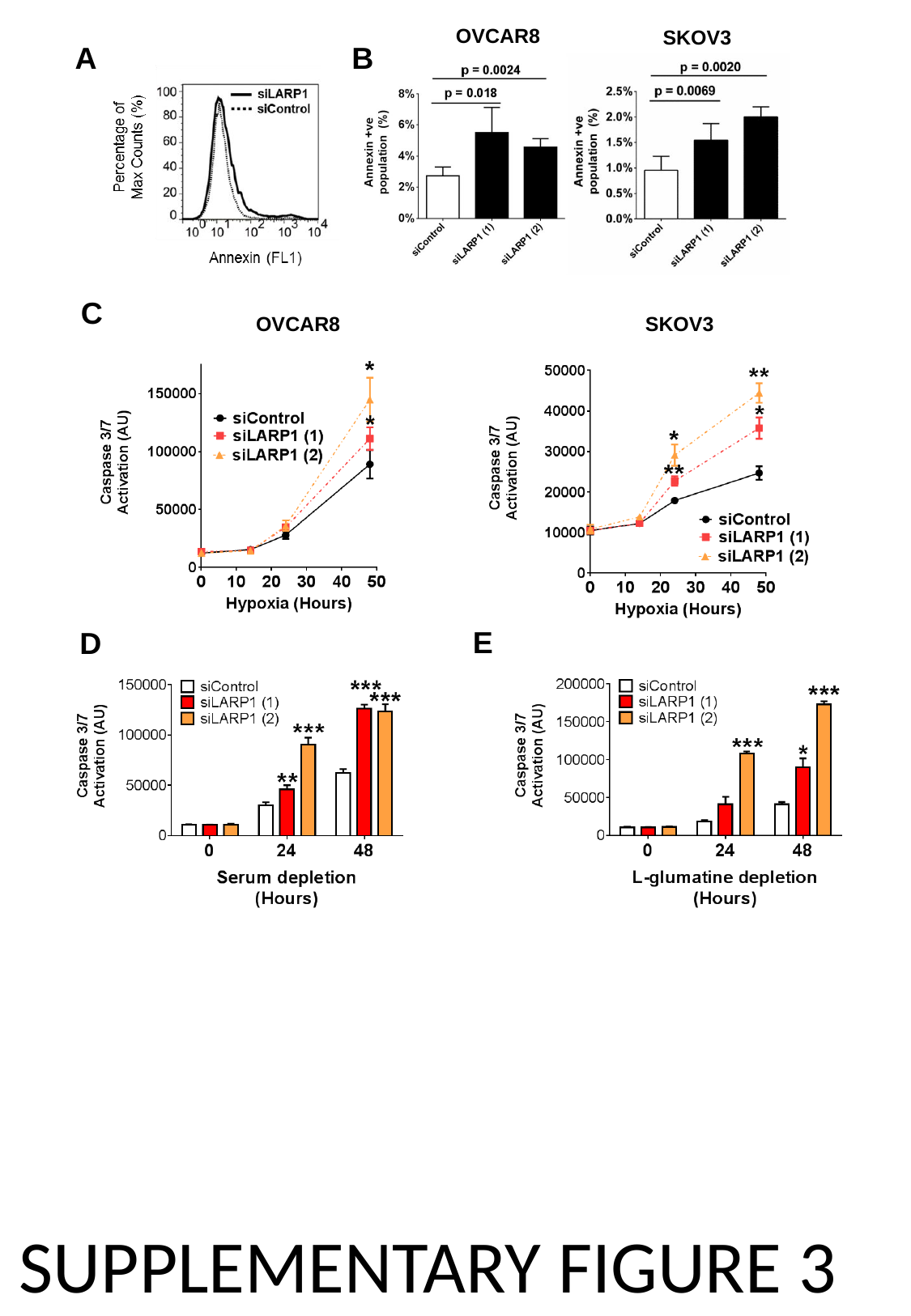

OVCAR8
SKOV3
A
B
C
OVCAR8
SKOV3
E
D
SUPPLEMENTARY FIGURE 3

## Slide 4
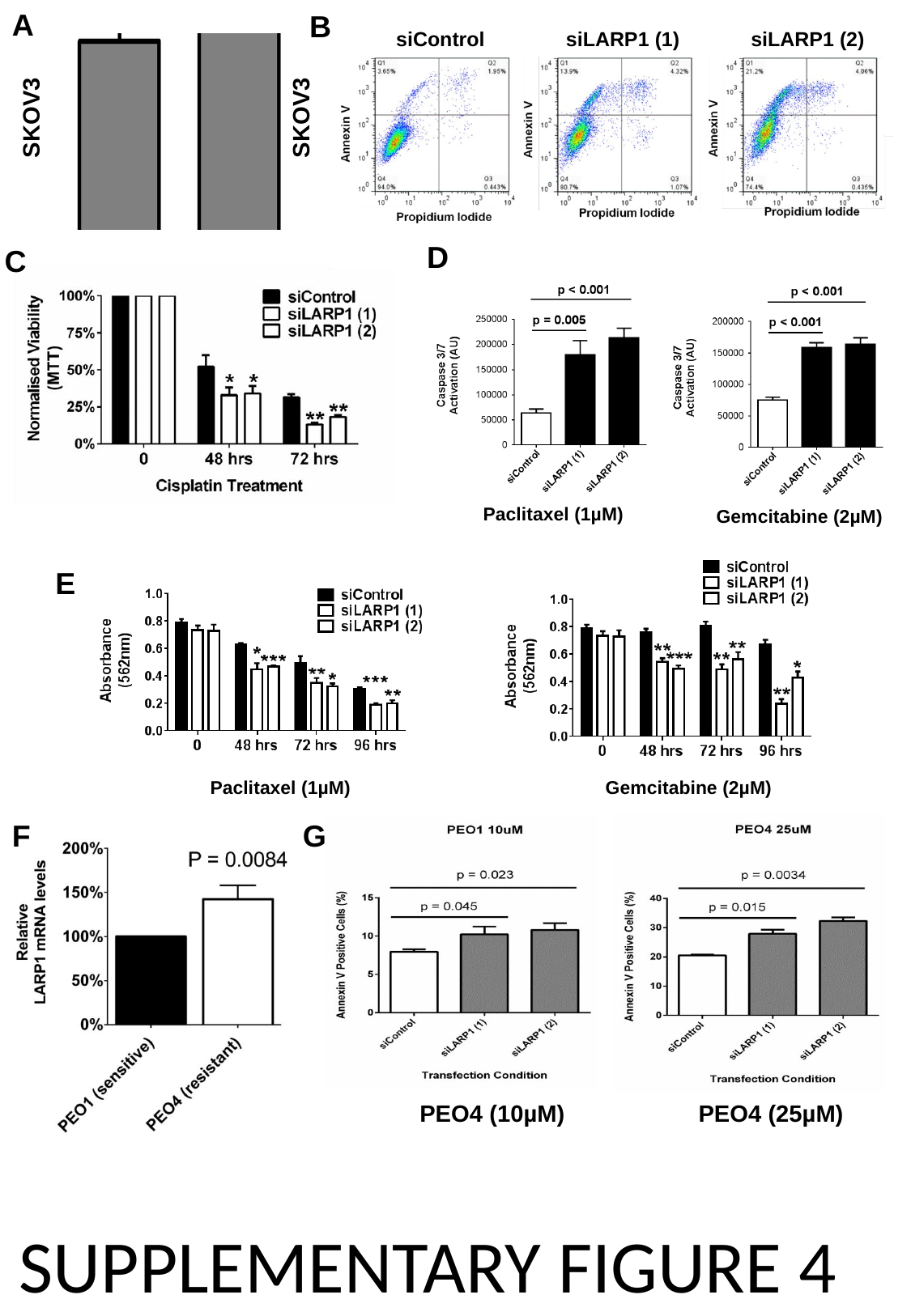

A
B
siControl
siLARP1 (1)
siLARP1 (2)
SKOV3
SKOV3
D
C
Paclitaxel (1µM)
Gemcitabine (2µM)
E
Paclitaxel (1µM)
Gemcitabine (2µM)
F
G
PEO4 (25µM)
PEO4 (10µM)
SUPPLEMENTARY FIGURE 4

## Slide 5
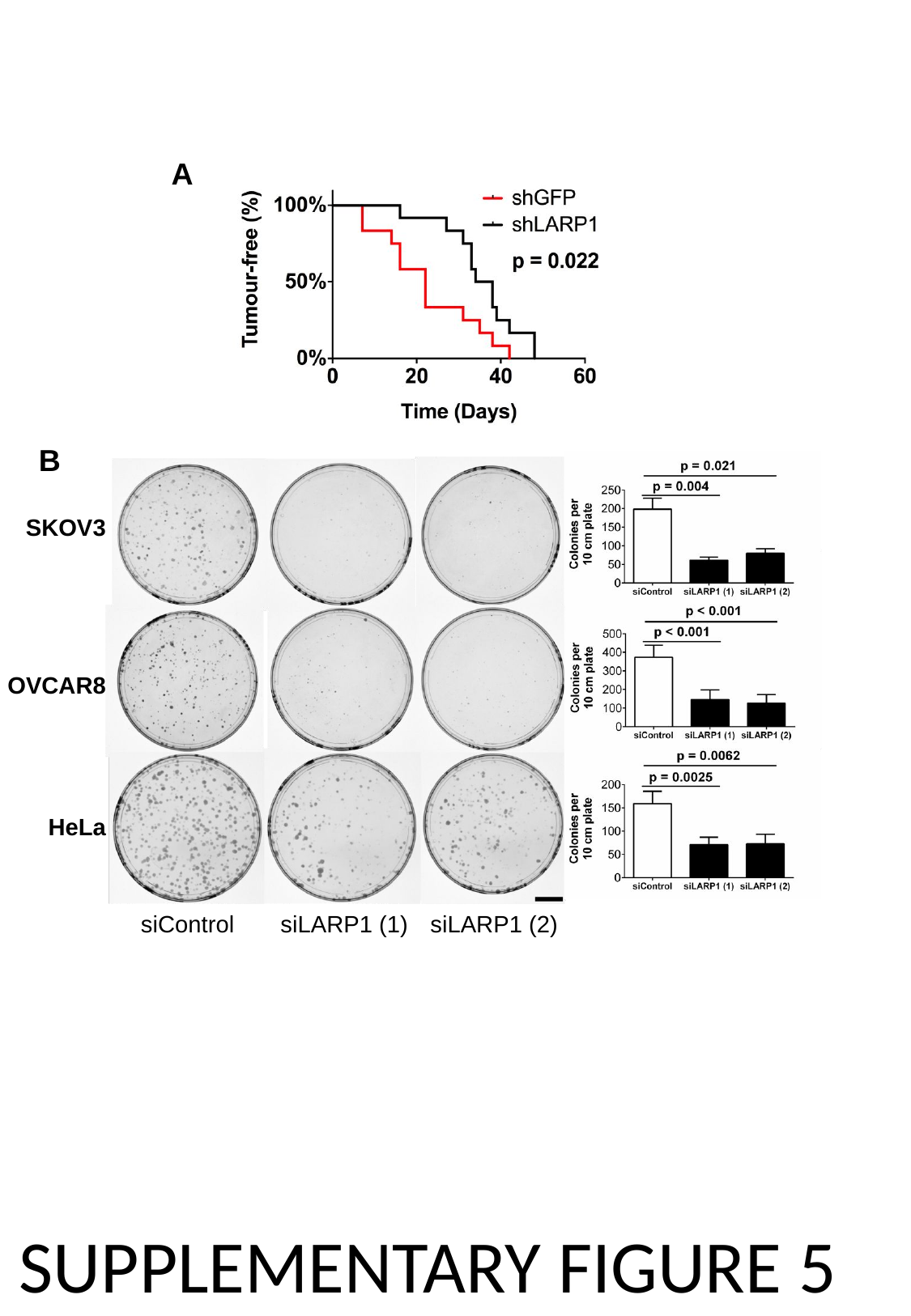

A
B
SKOV3
OVCAR8
HeLa
siControl
siLARP1 (1)
siLARP1 (2)
SUPPLEMENTARY FIGURE 5

## Slide 6
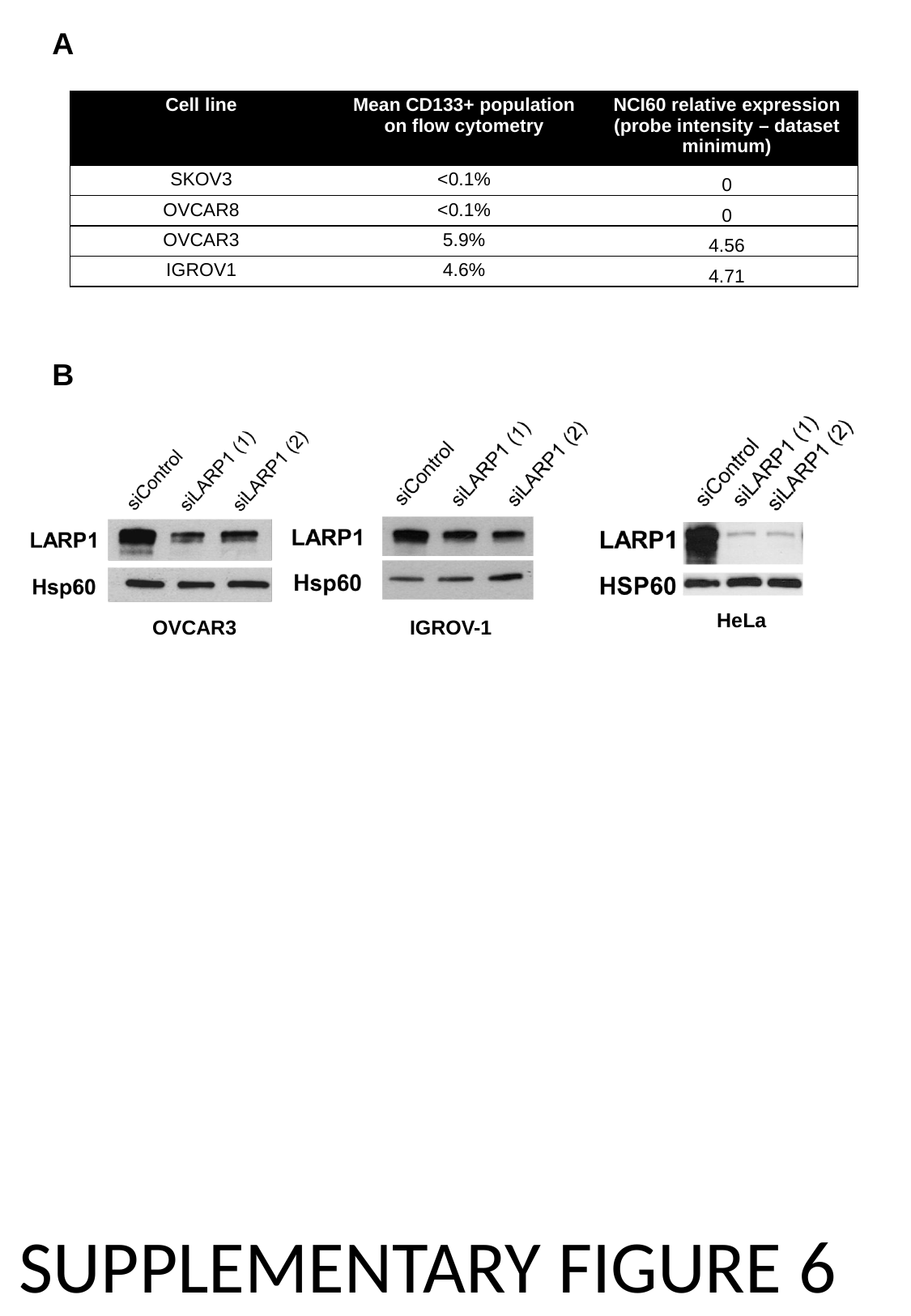

A
| Cell line | Mean CD133+ population on flow cytometry | NCI60 relative expression (probe intensity – dataset minimum) |
| --- | --- | --- |
| SKOV3 | <0.1% | 0 |
| OVCAR8 | <0.1% | 0 |
| OVCAR3 | 5.9% | 4.56 |
| IGROV1 | 4.6% | 4.71 |
B
HeLa
OVCAR3
IGROV-1
SUPPLEMENTARY FIGURE 6

## Slide 7
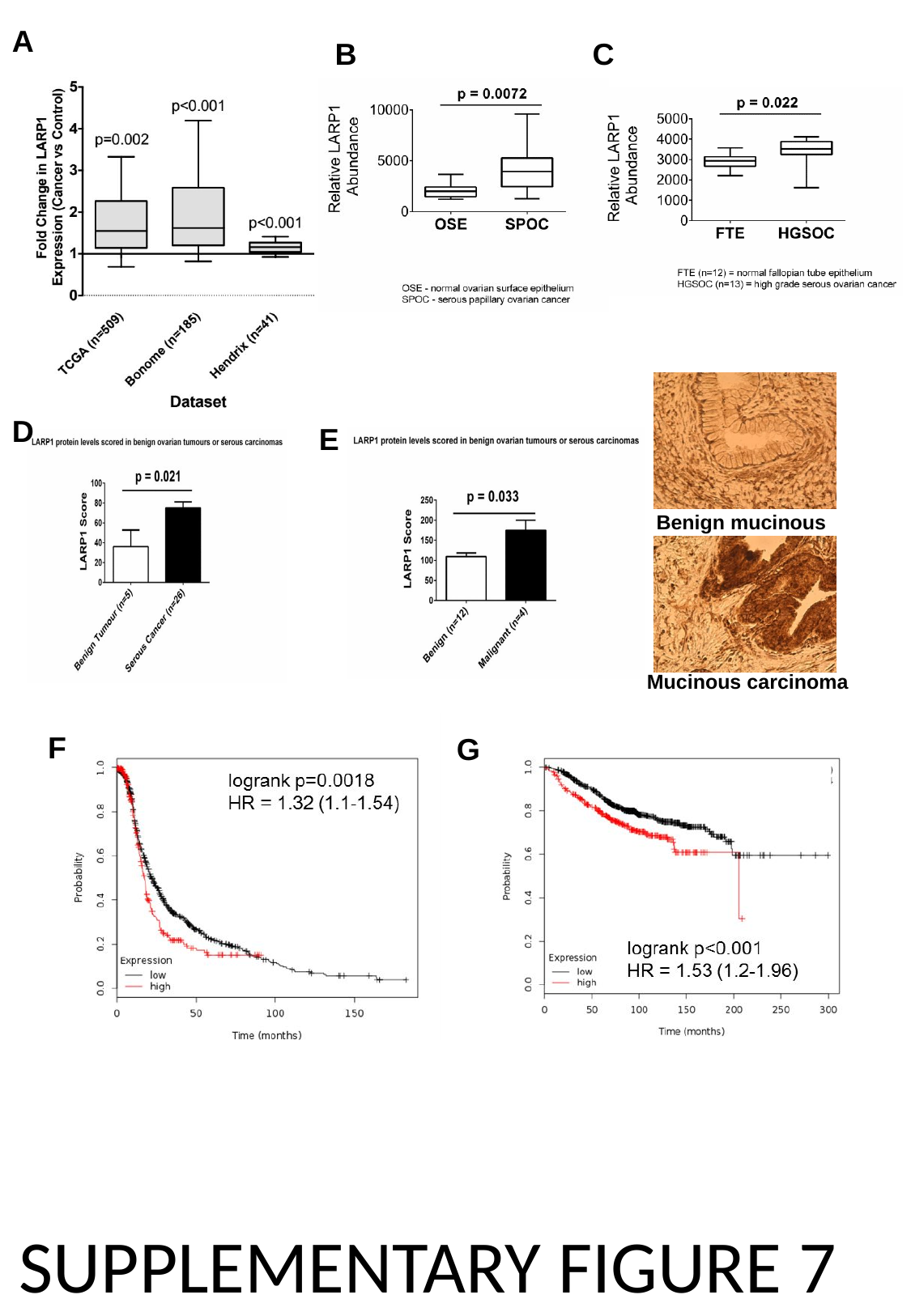

A
B
C
E
Benign mucinous
Mucinous carcinoma
D
F
G
SUPPLEMENTARY FIGURE 7
